# Pancreatic stone protein versus conventional biomarkers for prognostic discrimination in sepsis: a meta-analysis of paired AUC comparisons

**DOI:** 10.1186/s40635-026-00990-6

**Published:** 2026-10-20

**Authors:** Gabriele Melegari, Valerio Valente, Federica Arturi, Ilaria Pacini, Eros Pilia, Alessandro Belletti, Andrea Tognù, Elisabetta Bertellini, Alberto Barbieri

**Affiliations:** 1 Department of Anesthesia and Intensive Care, Azienda Ospedaliero-Universitaria Policlinico di Modena, Via del Pozzo 71, Modena, 41124 Italy; 2 School of Anesthesia and Intensive Care, University of Modena and Reggio Emilia, Modena, Italy; 3 Department of Anaesthesia and Intensive Care, IRCCS Istituto Ortopedico Rizzoli, Bologna, Italy; 4 Department of Medical Science and Public Health, University of Cagliari, Cagliari, Italy; 5 Department of Cardio-Thoracic Anaesthesia and Intensive Care IRCCS San Raffaele Scientific Institute, Milan, Italy; 6 Department of Anaesthesia and Intensive Care, ASST Gaetano Pini-CTO, Milan, Italy; 7 School of Medcine, University of Modena and Reggio Emilia, Modena, Italy

**Keywords:** Sepsis, Septic shock, Pancreatic stone protein, Procalcitonin, C-reactive protein, Biomarkers, Prognosis, Meta-analysis, AUC, Critical care

## Abstract

**Background:**

Pancreatic stone protein (PSP) has been proposed as a prognostic biomarker in sepsis, but its comparative performance versus C-reactive protein (CRP) and procalcitonin (PCT) remains uncertain.

**Methods:**

We performed a meta-analysis of direct within-study paired AUC comparisons between PSP and CRP and/or PCT in critically ill adults with sepsis, septic shock, or acute infection-related conditions in whom sepsis development or clinically relevant adverse outcomes were prospectively evaluated. Differences in AUC (ΔAUC) were pooled using random-effects models. The principal conservative analysis included one comparison per independent cohort and comparator.

**Results:**

Seven studies comprising 803 unique participants contributed 21 paired comparisons. In the principal conservative analysis, PSP showed a higher prognostic AUC than CRP, with a pooled ΔAUC of 0.179 (95% CI 0.095–0.262; *p* < 0.001), although between-cohort heterogeneity was substantial (I² = 81.65%). Compared with PCT, the pooled ΔAUC was 0.153 (95% CI 0.114–0.193; *p* < 0.001), with no observed heterogeneity (I² = 0.00%). The mortality-only sensitivity analysis showed a similar pooled effect for PSP versus CRP (ΔAUC 0.17, 95% CI 0.08–0.26), with substantial heterogeneity persisting, and for PSP versus PCT (ΔAUC 0.153, 95% CI 0.114–0.193), with no observed heterogeneity. Exclusion of the clinically distinct intra-abdominal infection cohort (Michailides et al.) likewise did not materially change the PSP-versus CRP estimate (ΔAUC 0.166, 95% CI 0.077–0.256). The direction and approximate magnitude of the pooled effects were also preserved across alternative assumptions for the within-study correlation.

**Conclusions:**

Available comparative evidence suggests that PSP may have greater prognostic discrimination than CRP and PCT. The findings were broadly maintained across sensitivity analyses, but the limited number of independent cohorts and substantial heterogeneity in the CRP comparison warrant caution. Further prospective studies are required to confirm these findings and establish their clinical relevance.

**Trial registration:**

This systematic review was prospectively registered in the International Prospective Register of Systematic Reviews (PROSPERO; CRD420261340301).

**Graphical abstract:**

**Figure:**
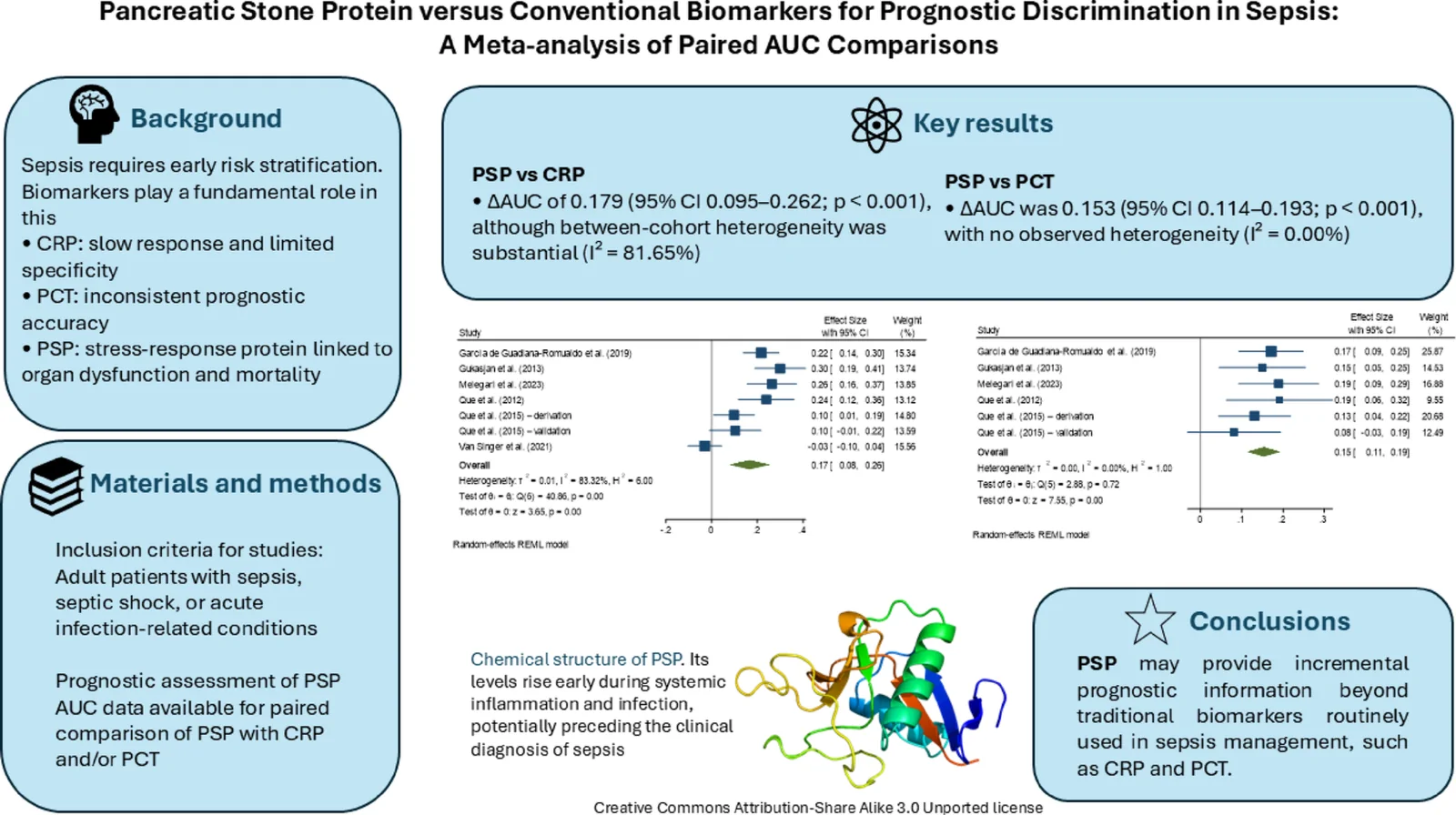

**Supplementary Information:**

The online version contains supplementary material available at https://doi.org/10.1186/s40635-026-00990-6.

## Background

Sepsis and septic shock continue to be major causes of death among critically ill patients around the world, imposing a significant burden on healthcare systems. Although there have been advancements in supportive care and antimicrobial treatments, early risk assessment and prompt treatment decisions remain difficult [1]. In this context, biomarkers have been extensively studied to aid in the diagnosis, prognosis, and therapeutic monitoring of sepsis. Currently, C-reactive protein (CRP) and procalcitonin (PCT) are the most commonly used biomarkers in clinical practice. However, both have significant limitations. CRP is slow to respond and lacks specificity for infections, while PCT, although more closely associated with infections, can be affected by non-infectious inflammatory conditions and does not consistently provide reliable prognostic information. Consequently, neither biomarker alone meets the criteria for an ideal prognostic marker in sepsis [2–4].

Pancreatic Stone Protein (PSP), also known as regenerating protein-1α (Reg1α), has recently emerged as a promising biomarker in critically ill patients. Initially described in the context of pancreatic disease, PSP is now recognized as a stress-response protein involved in innate immune modulation [5, 6]. Experimental and clinical data suggest that PSP levels rise early during systemic inflammation and infection, potentially preceding the clinical diagnosis of sepsis [7]. Moreover, PSP has been associated with organ dysfunction severity and mortality across different critical care populations.

Observational prognostic studies have reported superior prognostic performance of PSP compared with traditional biomarkers [8, 9]. Additional early studies evaluating PSP in paediatric or purely diagnostic settings are also available [10, 11]; these data are cited only as background evidence supporting the biological and clinical interest in PSP and were not included in the quantitative synthesis, which was restricted to adult prognostic studies in adults with sepsis, septic shock, or acute infection-related conditions. Nevertheless, results across studies remain heterogeneous, and direct quantitative comparisons between PSP and standard biomarkers within the same patient populations are limited. In particular, the relative prognostic discrimination of PSP versus CRP and PCT has not been systematically synthesized using paired comparisons.

## Aim of the study

Therefore, the aim of this study was to perform a meta-analysis of direct intra-study paired comparisons to determine whether Pancreatic Stone Protein (PSP) provides greater prognostic discrimination than C-reactive protein (CRP) and procalcitonin (PCT) for clinically relevant adverse outcomes in critically ill adults with sepsis, septic shock, or severe infection. The primary effect measure was the difference in the area under the receiver operating characteristic curve (ΔAUC) between PSP and each comparator biomarker. In addition to the overall comparative assessment, a mortality-only analysis restricted to baseline biomarker measurements was undertaken to evaluate whether the comparative prognostic discrimination of PSP was preserved for a more homogeneous and clinically definitive endpoint.

## Materials and methods

### Study design and reporting standards

This study conducted a systematic review and meta-analysis of prognostic accuracy studies evaluating Pancreatic Stone Protein (PSP) in sepsis and septic shock. The review was conducted and reported in accordance with the PRISMA (Preferred Reporting Items for Systematic Reviews and Meta-Analyses) guidelines and adhered to recommended methods for meta-analyses of prognostic accuracy [12]. The quantitative synthesis focused on direct within-study paired comparisons of AUCs between PSP and conventional biomarkers to assess the comparative prognostic discrimination of PSP. Additional methodological details are provided in the Supplementary Material. The review protocol was prospectively registered in PROSPERO (CRD420261340301) before study selection and data extraction commenced. Where methodological refinements were introduced during the quantitative synthesis to address characteristics of the available evidence, these are explicitly identified in the Methods and acknowledged as protocol deviations where appropriate.

### Definition of outcomes

Critical outcomes included mortality (in-hospital, 28-day, or longer-term mortality), organ failure, ICU readmission, and need for therapeutic escalation. Baseline biomarker measurements (T0) were defined as the earliest sampling time point reported in each study (hospital admission, ICU admission, or time of sepsis diagnosis). Baseline measurements were prioritized in the principal conservative analysis. When multiple eligible outcomes were reported within the same cohort, a single outcome was selected according to the clinical hierarchy described below.

### Search strategy

A structured literature search was conducted in PubMed, EMBASE, and Scopus from database inception to 13 March 2026. The search used database-specific combinations of free-text terms and synonyms related to pancreatic stone protein, including “pancreatic stone protein”, “pancreatic stone protein/regenerating protein”, “PSP/reg”, “REG1A”, “REG1 alpha”, and “lithostathine”. A sensitive biomarker-based search strategy was used, with clinical eligibility criteria related to sepsis, septic shock, and acute infection-related conditions applied during study screening. Reference lists of relevant reviews and eligible articles were also screened to identify additional studies. No restrictions were applied to study design. Only studies involving human participants and published in English were considered. The complete database-specific search strategies are provided in the Supplementary Material.

### Eligibility criteria

Studies were eligible if they: (1) included adults with sepsis or septic shock, or adults with an acute infection-related condition in whom sepsis development or clinically relevant adverse outcomes were prospectively evaluated; (2) reported the prognostic performance of PSP together with C-reactive protein (CRP) and/or procalcitonin (PCT) measured in the same patient population; (3) provided paired AUC estimates for PSP and at least one comparator biomarker for an eligible prognostic outcome; and (4) reported, or allowed derivation of, the corresponding 95% confidence intervals. Studies exclusively evaluating paediatric populations or the diagnostic performance of biomarkers without assessment of subsequent clinical outcomes were excluded.

The inclusion of adults with an acute infection-related condition, in addition to patients with established sepsis or septic shock at enrolment, represents a post-registration clarification of the population criterion. This clarification was required because one eligible prospective study enrolled patients with intra-abdominal infection and evaluated subsequent sepsis-related adverse outcomes. This deviation from the narrower wording of the PROSPERO record is explicitly acknowledged.

### Unit of analysis

The study-condition unit was defined as a paired comparison between PSP and a comparator biomarker (CRP or PCT) within the same patient population, for a given outcome and timepoint. This approach preserved the within-study paired comparative prognostic information and avoided biases associated with indirect comparisons. Because multiple study-condition units derived from the same cohort may share participants, biomarker measurements, or outcome events, these units were not assumed to be statistically independent. Accordingly, the unit of analysis for the principal conservative analysis was the independent cohort, with only one comparison retained per independent cohort and comparator. All eligible paired comparisons were retained in a broader supportive analysis.

### Data extraction

Data extraction was performed using a predefined template. For each study-condition unit, the following variables were collected: AUC values for PSP and comparator biomarkers, corresponding 95% confidence intervals, clinical setting, population characteristics, outcome definition, timing of biomarker measurement, and assay type, when available. Confidence intervals reported graphically or textually were converted into numeric lower and upper bounds. All values were standardized before analysis.

### Estimation of standard errors

When standard errors (SEs) of the AUC estimates were not directly reported, they were derived from the corresponding 95% confidence intervals using the normal approximation:

$$\mathrm{S}\mathrm{E} = (\mathrm{u}\mathrm{p}\mathrm{p}\mathrm{e}\mathrm{r}\:95 \%\:\mathrm{C}\mathrm{I} - \mathrm{l}\mathrm{o}\mathrm{w}\mathrm{e}\mathrm{r}\:95 \% \:\mathrm{C}\mathrm{I}) / 3.92$$

This approach assumes an approximately symmetric normal sampling distribution of AUC estimates and was considered appropriate because the reported AUC values were generally well within the theoretical boundaries of 0 and 1. When standard errors were directly reported, the original values were used. This approach was applied uniformly across studies to ensure comparability.

### Statistical analysis

The primary effect size was the difference in AUC (ΔAUC) between PSP and each comparator biomarker: ΔAUC = AUC(PSP) – AUC(comparator). A positive ΔAUC indicated superior prognostic discrimination for PSP. Variance estimation for paired comparisons. Because PSP and comparator biomarkers were measured in the same individuals, AUC estimates were considered correlated. Accordingly, the variance of the paired difference in AUC (ΔAUC) was estimated as:

$$\begin{aligned}\mathrm{V}\mathrm{a}\mathrm{r}\left(\mathrm{A}\mathrm{U}\mathrm{C}\right) & =\mathrm{V}\mathrm{a}\mathrm{r}(\mathrm{A}\mathrm{U}\mathrm{C}_{1}) +\mathrm{V}\mathrm{a}\mathrm{r}(\mathrm{A}\mathrm{U}\mathrm{C}_{2})\\ & \quad - 2(\mathrm{S}\mathrm{E}_{1}\:\times\:\:\mathrm{S}\mathrm{E}_{2}), \end{aligned}$$

where SE₁ and SE₂ denote the standard errors of the respective AUC estimates for PSP and the comparator biomarker and ρ denotes the assumed within-study correlation between the paired AUC estimates. When standard errors of the AUC estimates were not directly reported, they were derived from the corresponding 95% confidence intervals using the normal approximation: SE = (upper 95% CI − lower 95% CI) / 3.92. This approximation assumes a roughly symmetric normal sampling distribution for the AUC estimate and was used only when standard errors were unavailable from the original study. In the absence of empirical covariance data, an intermediate correlation coefficient of ρ = 0.60 was selected as a pragmatic analytical assumption for variance estimation. Sensitivity analyses explored alternative values (ρ = 0.30 and ρ = 0.80). Random-effects meta-analyses were conducted separately for PSP vs. CRP and PSP vs. PCT. Random-effects models were chosen to account for clinical and methodological heterogeneity. Between-study variance was estimated using Restricted Maximum Likelihood (REML). Statistical heterogeneity was assessed using Cochran’s Q and quantified using I² statistics. Heterogeneity (I²) was calculated using the implementation provided in Stata’s meta-analysis framework. All analyses were conducted using Stata (StataCorp LLC, College Station, TX) with dedicated meta-analysis commands.

### Risk of bias and applicability assessment

Because no specific risk-of-bias instrument had been prespecified in the PROSPERO registration, the assessment was revised post hoc using the QUADAS-C framework, an extension of QUADAS-2 developed for comparative accuracy studies. QUADAS-C was selected because the included studies directly compared the prognostic discrimination of PSP with CRP and/or PCT within the same participants. A review-specific prognostic adaptation was applied, replacing the diagnostic “reference standard” construct with ascertainment of the prognostic outcome, while retaining the original comparative structure of the tool.

Risk of bias was first considered for each biomarker separately using the relevant QUADAS-2 signalling questions and was then assessed for the direct comparison between PSP and the comparator biomarker using QUADAS-C. Four comparative domains were evaluated: patient selection, conduct and interpretation of the index tests, outcome ascertainment, and flow and timing. The assessment specifically considered consecutive enrolment, inappropriate exclusions, paired measurement of the biomarkers, blinding, data-driven threshold selection, comparability of sampling times, independence and objectivity of outcome ascertainment, completeness of follow-up, and biomarker-specific missing data. Comparative risk of bias judgments were classified as low, high, or unclear. A low-risk judgment required sufficient information to exclude a relevant source of comparative bias; an unclear judgment was assigned when reporting was insufficient; and a high-risk judgment was assigned when a plausible source of comparative bias was identified. Applicability concerns were evaluated separately for patient population, biomarker measurement, and prognostic outcome, in accordance with the QUADAS-2 framework. Review-specific signalling questions, decision rules, and study-level rationales are provided in the Supplementary Material.

### Principal conservative analysis

To address the potential non-independence of multiple study-condition units derived from the same cohort, the principal conservative analysis for inference was restricted to one comparison per independent cohort and comparator. Baseline measurements (T0) were prioritized. When multiple outcomes were available within the same cohort, only one comparison was retained according to a clinical hierarchy: mortality, organ failure, therapeutic escalation, and ICU readmission. This approach was intended to minimize potential double counting arising from shared participants, biomarker measurements, and outcome events while preserving the paired within-cohort comparison between PSP and the comparator biomarker. The broader analysis including all eligible paired comparisons was retained as a supportive analysis to describe the full comparative evidence available from the included studies.

### Sensitivity analyses

Sensitivity analyses were conducted to evaluate the influence of the assumed within-study correlation between paired AUC estimates. Because PSP and the comparator biomarkers were measured in the same patients and evaluated against the same outcome, a positive correlation between AUC estimates was expected. However, empirical covariance data were unavailable and could not be reconstructed from the published studies. An intermediate correlation coefficient (ρ = 0.60) was therefore selected for variance estimation, and the stability of the results was explored using lower (ρ = 0.30) and higher (ρ = 0.80) values [13]. The choice of ρ remains an analytical assumption and is acknowledged as a limitation. Analyses were conducted separately for the CRP and PCT comparators. Additional sensitivity analyses were performed by restricting the analysis to baseline (T0) mortality outcomes and by excluding the Michailides et al. cohort. As supportive analyses, pooled estimates were also calculated using all eligible paired comparisons and after restriction to baseline (T0) measurements.

## Results

### Study selection

**Fig. 1:**
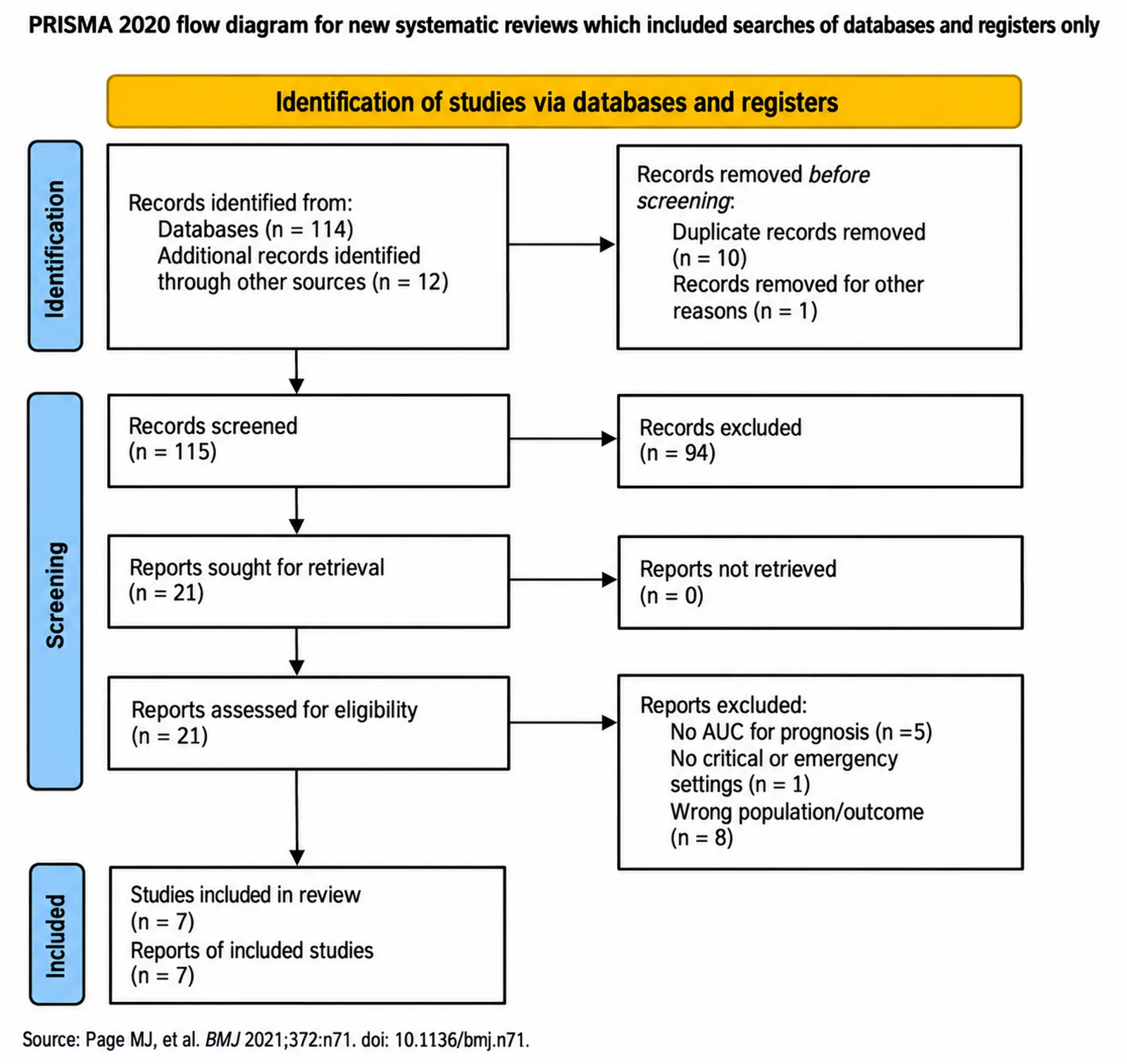
The study selection process (PRISMA flow diagram)

The literature search identified 126 records, of which 114 were retrieved from electronic databases and 12 from other sources. After removal of 10 duplicates and 1 record excluded for other reasons, 115 records underwent title and abstract screening. Of these, 94 were excluded based on predefined eligibility criteria. Twenty-one full-text articles were assessed for eligibility. Fourteen studies were excluded at the full-text stage for the following reasons: lack of prognostic AUC data (*n* = 5), non-critical or non-emergency clinical settings (*n* = 1), and wrong population or outcome definition (*n* = 8). Ultimately, 7 studies met all inclusion criteria and were included in the quantitative synthesis. The study selection process is summarized in Fig. 1 (PRISMA flow diagram) [8, 9, 14–18]. The main characteristics of the included studies are presented in Table 1. Seven studies comprising 803 unique participants contributed 21 paired intra-study comparisons. Of these, 19 corresponded to baseline (T0) measurements, including 11 PSP versus CRP comparisons and 8 PSP versus PCT comparisons. Two additional comparisons were derived from non-baseline timepoints. Because multiple comparisons originating from the same cohort may share participants, biomarker measurements, or outcome events, they were not assumed to be statistically independent. Accordingly, the principal conservative analysis for inference was restricted to one comparison per independent cohort and comparator, as specified in the Methods. Mortality was the most frequently evaluated endpoint, while ICU readmission, therapeutic escalation, and multi-organ failure were also represented in the broader evidence base.

**Table 1:** the main characteristics of the included studies

Study (Year)	Clinical setting / Population	*N* (events)	Outcome	Timepoint	PSP assay / platform	PSP AUC (95% CI)	CRP AUC (95% CI)	PCT AUC (95% CI)
García de Guadiana-Romualdo et al. 2019	ICU patients with sepsis	122 (11 deaths)	28-day mortality	Admission (T0)	Sandwich isoform-specific ELISA	0.724 (0.636–0.801)	0.507 (0.415–0.599)	0.553 (0.460–0.643)
Gukasjan et al. 2013	Postoperative patients with secondary peritonitis admitted to ICU	91 (23 deaths)	ICU mortality	ICU admission (< 3 h)	Isoform-specific sandwich ELISA	0.78 (0.67–0.88)	0.48 (0.35–0.62)	0.63 (0.51–0.76)
Gukasjan et al. 2013	Same cohort as above†	91 (19 with multiorgan failure)	Multi-organ failure	ICU admission (< 3 h)	Isoform-specific sandwich ELISA	0.75 (0.64–0.85)	0.53 (0.40–0.65)	0.71 (0.60–0.82)
Melegari et al. 2023	Mechanically ventilated ICU patients with COVID-19	21 (8 deaths)	90-day mortality	Baseline (T0)	Nanofluidic fluorescent immunoassay, abioSCOPE^®^ IVD	0.836 (0.735–0.937)	0.572 (0.439–0.705)	0.647 (0.533–0.761)
Michailides et al. 2023	Adults admitted with intra-abdominal infection	40; readmission data available in 36 (14 readmissions)	30-day readmission	Within 24 h of admission	Whole-blood point-of-care measurement, abioSCOPE^®^	0.734 (0.564–0.905)‡	0.628 (0.436–0.820)	—
Michailides et al. 2023	Same cohort as above†	40 (treatment-escalation event count: see footnote §)	Need for therapeutic escalation	Within 24 h of admission	Whole-blood point-of-care measurement, abioSCOPE^®^	0.862 (0.748–0.976)	0.575 (0.383–0.767)	—
Que et al. 2012	ICU patients with severe sepsis or septic shock	107 (22 deaths; 20.5%)	In-hospital mortality	Within 24 h of ICU admission	Isoform-specific ELISA	0.651 (0.515–0.787)	0.412 (0.275–0.550)	0.460 (0.310–0.611)
Que et al. 2012	Septic-shock subgroup of the same cohort¶	74 (19 deaths; 25.7%)	In-hospital mortality	Within 24 h of ICU admission	Isoform-specific ELISA	0.652 (0.507–0.796)	0.442 (0.288–0.597)	0.446 (0.284–0.607)
Que et al. 2015 – derivation cohort	ICU patients with severe sepsis or septic shock, Switzerland	158 (42 deaths; 27%)	In-hospital mortality	Within 24 h of ICU admission or onset of nosocomial sepsis	Plasma biomarker assays; methodological details reported in source e-Appendix 1	0.665 (0.571–0.758)	0.567 (0.461–0.673)	0.534 (0.433–0.636)
Que et al. 2015 – external-validation cohort	ICU patients with severe sepsis or septic shock, Brazil	91 (39 deaths; 43%)	In-hospital mortality	Within 24 h of ICU admission or onset of nosocomial sepsis	Plasma biomarker assays; methodological details reported in source e-Appendix 1	0.624 (0.492–0.753)	0.521 (0.398–0.643)	0.543 (0.422–0.663)
Van Singer et al. 2021	Consecutive adults presenting to the ED with RT-PCR-confirmed COVID-19	173 (12 deaths; 6.9%)	7-day mortality	ED presentation	Nanofluidic point-of-care immunoassay, abioSCOPE^®^	0.83 (0.74–0.92)	0.86 (0.79–0.93)||	—

**†** Multiple outcomes were evaluated within the same participant cohort; these rows therefore do not represent independent study populations. **‡** Michailides et al. report a PSP AUC of 0.734 (95% CI 0.564–0.905) for readmission in Table 2, whereas the accompanying Results text reports an AUC of 0.899 (95% CI 0.794–1.00). The value used in the quantitative dataset is shown in the table. **§** An internal discrepancy is present in the Michailides publication regarding treatment-escalation frequency: Table 1 reports 11/40 (27.5%), whereas the Results text states that treatment escalation was required in 29/40 patients. The published AUC estimates used in the meta-analysis are unaffected by this reporting discrepancy. **¶** The 74 patients with septic shock in Que et al. (2012) are a subgroup nested within the full 107-patient cohort and were therefore not treated as an independent cohort in the principal conservative analysis. || Van Singer et al. report a CRP AUROC of 0.83 (95% CI 0.79–0.93) in the main text, whereas Fig. 1A reports 0.86 (95% CI 0.79–0.93). The value 0.86 reported in the original ROC figure was used for the quantitative synthesis [15]. Abbreviations: PSP, pancreatic stone protein; CRP, C-reactive protein; PCT, procalcitonin; AUC, area under the receiver operating characteristic curve; CI, confidence interval; ICU, intensive care unit; ED, emergency department

### Mortality-only sensitivity analysis: CRP

**Fig. 2:**
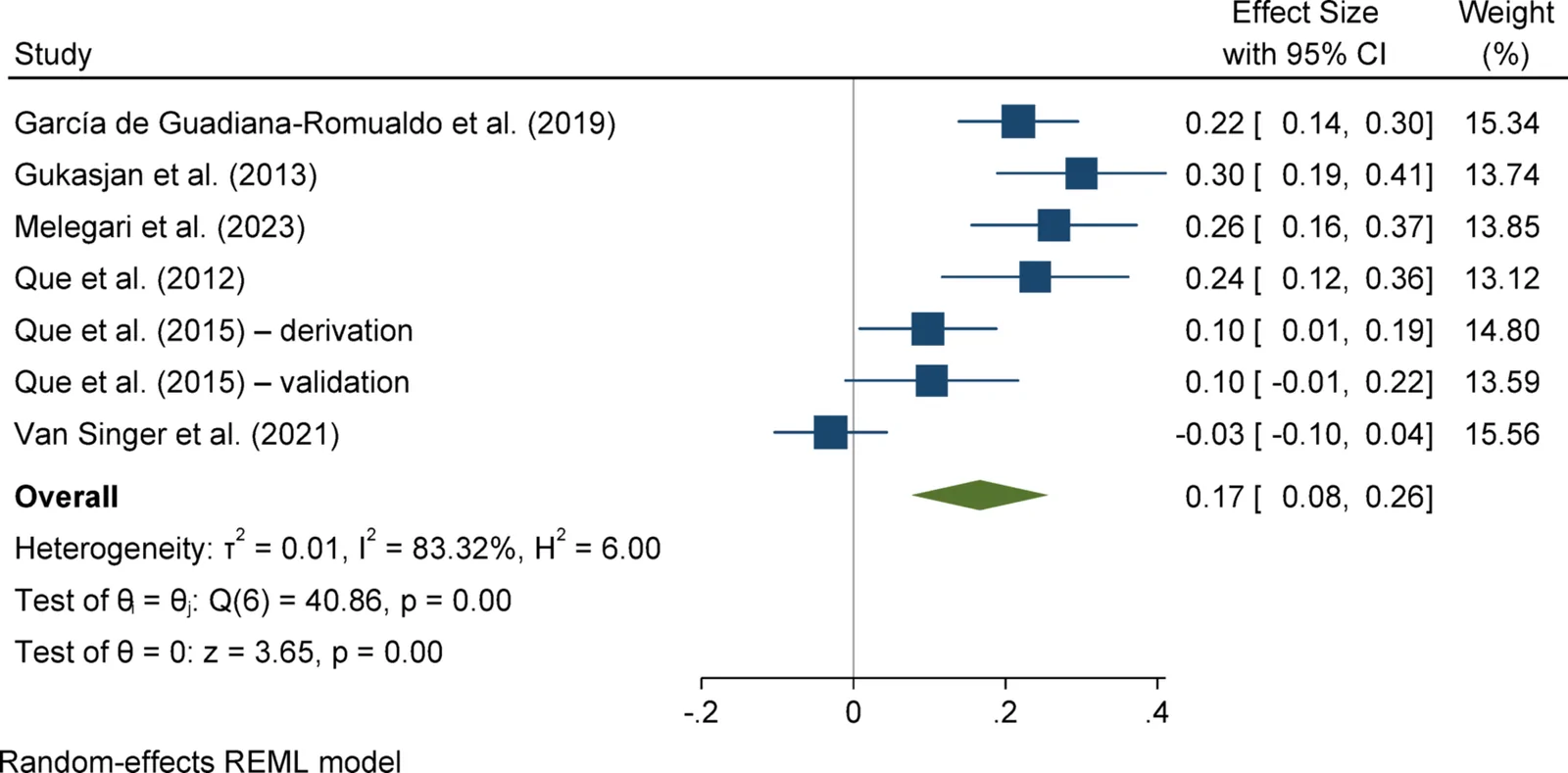
Mortality-only sensitivity analysis comparing the prognostic discrimination of pancreatic stone protein and C-reactive protein. Forest plot of paired differences in the area under the receiver operating characteristic curve (ΔAUC) between pancreatic stone protein (PSP) and C-reactive protein (CRP), restricted to baseline (T0) mortality outcomes and one comparison per independent cohort. Positive ΔAUC values indicate greater prognostic discrimination for PSP, whereas the vertical line at ΔAUC = 0 represents no difference between biomarkers

To assess whether the comparative prognostic discrimination of PSP was preserved for a more homogeneous and clinically definitive endpoint, an additional sensitivity analysis was restricted to mortality outcomes assessed at baseline (T0), including one comparison per independent cohort and comparator. For PSP versus CRP, seven independent cohorts were included. Random-effects meta-analysis using REML showed a pooled ΔAUC of 0.17 (95% CI 0.08–0.26; *p* < 0.001) in favour of PSP. Substantial between-cohort heterogeneity was observed (I² = 83.32%; τ² = 0.01; Q(6) = 40.86, *p* < 0.001). These findings indicate that the greater prognostic discrimination of PSP over CRP was maintained when the analysis was restricted to baseline mortality prediction, although considerable heterogeneity remained across cohorts (Fig. 2).

### Mortality-only sensitivity analysis: PCT

**Fig. 3:**
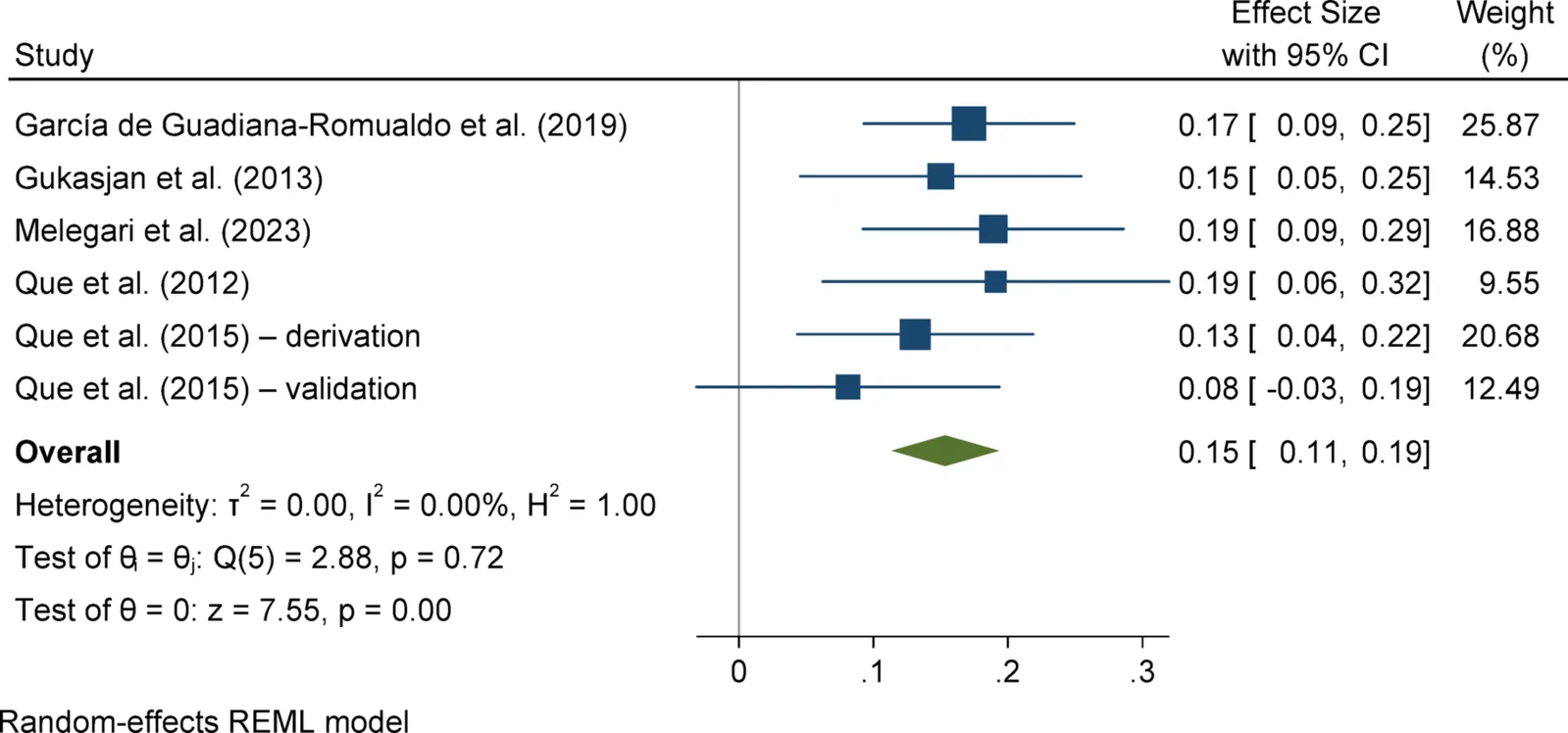
Mortality-only sensitivity analysis comparing the prognostic discrimination of pancreatic stone protein (PSP) and procalcitonin (PCT). Forest plot of paired differences in the area under the receiver operating characteristic curve (ΔAUC) between PSP and PCT, restricted to baseline (T0) mortality outcomes and one comparison per independent cohort. Because all six cohorts in the principal PSP-versus-PCT analysis report mortality as the outcome, this analysis is numerically identical to the principal conservative analysis shown in Fig. 5

In the mortality-only sensitivity analysis restricted to baseline (T0) measurements, six independent cohorts contributed to the comparison between PSP and PCT. Random-effects meta-analysis using REML showed a pooled ΔAUC of 0.153 (95% CI 0.114–0.193; *p* < 0.001) in favour of PSP. No between-cohort heterogeneity was observed (I² = 0.00%; τ² = 0.0000; Q(5) = 2.88, *p* = 0.718) (Fig. 3).

### Principal conservative analysis

**Fig. 4:**
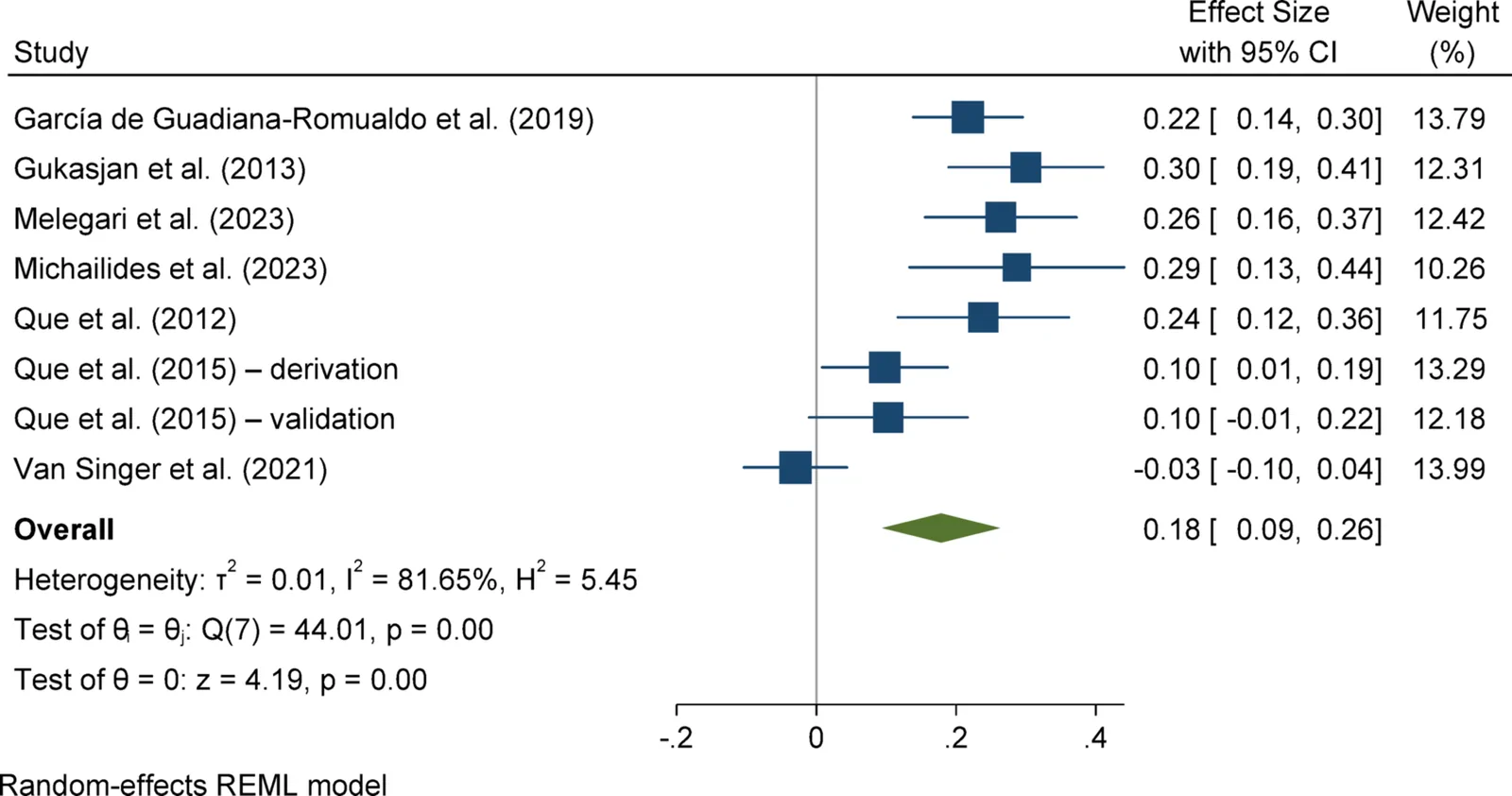
Principal conservative analysis comparing the prognostic discrimination of pancreatic stone protein (PSP) and C-reactive protein (CRP). Forest plot of paired differences in the area under the receiver operating characteristic curve (ΔAUC) between PSP and CRP. The analysis was restricted to baseline (T0) measurements and one comparison per independent cohort and comparator, selected according to the specified clinical hierarchy

In the principal conservative analysis, restricted to baseline measurements and one comparison per independent cohort and comparator according to the outcome hierarchy, eight independent cohorts contributed to the PSP versus CRP comparison. Random-effects meta-analysis using REML showed a pooled ΔAUC of 0.179 (95% CI 0.095–0.262; *p* < 0.001) in favour of PSP. Substantial between-cohort heterogeneity was observed (I² = 81.65%; τ² = 0.0116; Q(7) = 44.01, *p* < 0.001) (Fig. 4).

**Fig. 5:**
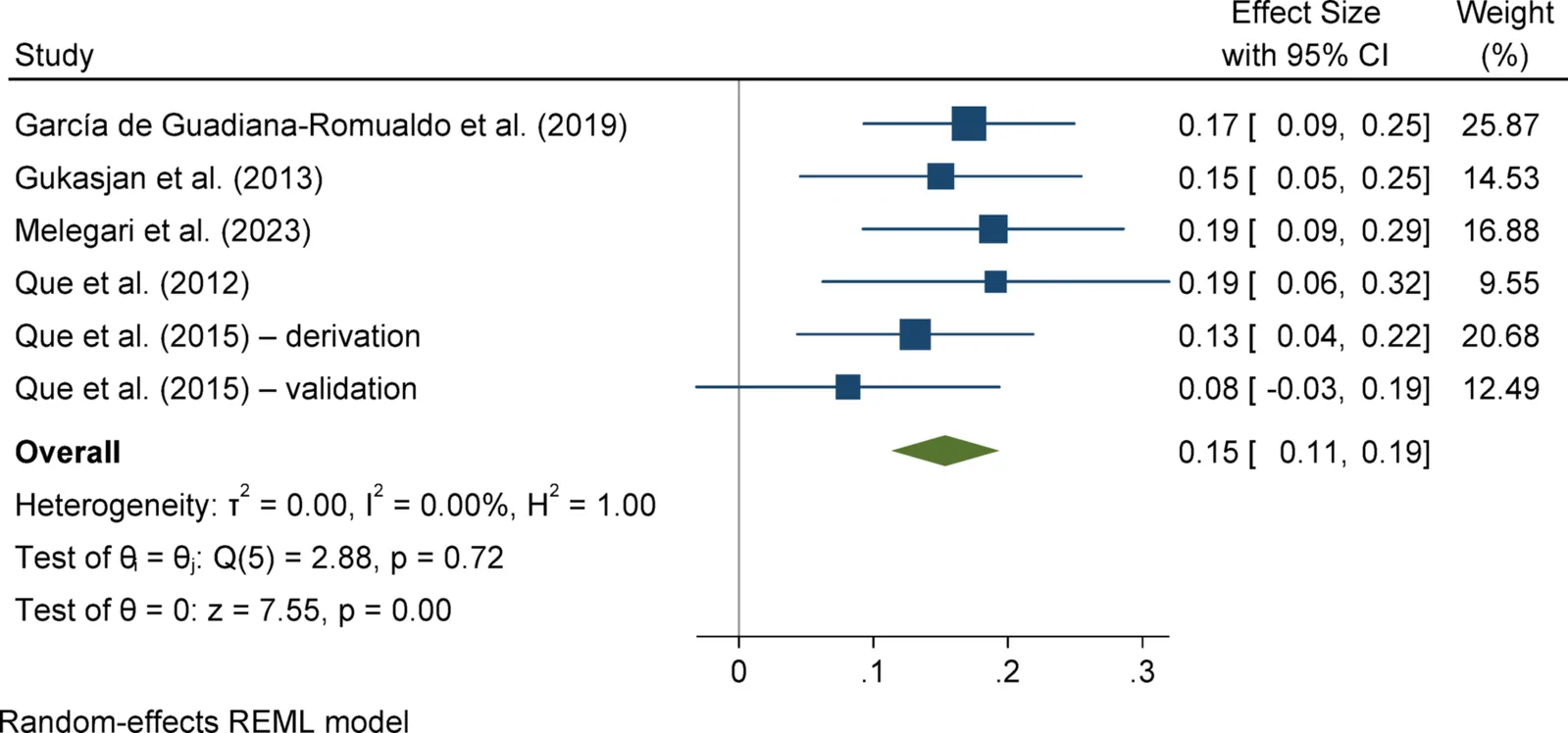
Principal conservative analysis comparing the prognostic discrimination of pancreatic stone protein (PSP) and procalcitonin (PCT). Forest plot of paired differences in the area under the receiver operating characteristic curve (ΔAUC) between PSP and PCT. The analysis was restricted to baseline (T0) measurements and one comparison per independent cohort and comparator, selected according to the specified clinical hierarchy

For the PSP versus PCT comparison, six independent cohorts were included. The pooled ΔAUC was 0.153 (95% CI 0.114–0.193; *p* < 0.001) in favour of PSP. No between-cohort heterogeneity was observed (I² = 0.00%; τ² = 0.00; Q(5) = 2.88, *p* = 0.72) (Fig. 5).

#### Sensitivity analysis excluding Michailides et al

**Fig. 6:**
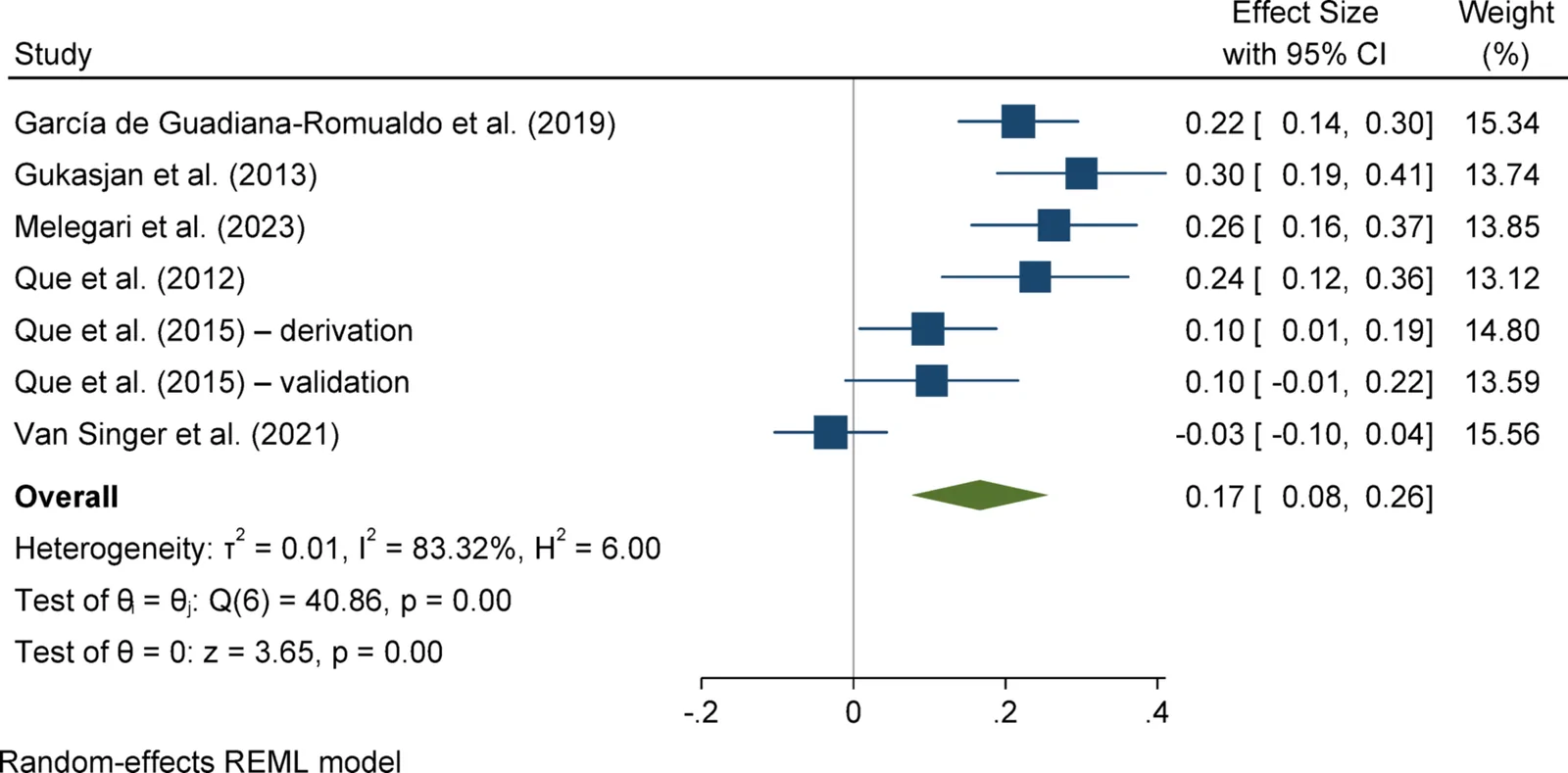
Sensitivity analysis excluding the Michailides et al. cohort: PSP versus CRP. Forest plot of paired differences in the area under the receiver operating characteristic curve (ΔAUC) between pancreatic stone protein (PSP) and C-reactive protein (CRP), after exclusion of the Michailides et al. cohort from the principal conservative analysis. Because Michailides et al. does not contribute a mortality outcome to the CRP comparison, the seven remaining cohorts are identical to those in the mortality-only analysis shown in Fig. 2

To assess the potential influence of the Michailides et al. cohort on the principal conservative analysis, the PSP versus CRP meta-analysis was repeated after excluding this cohort. The pooled effect remained similar, with a ΔAUC of 0.166 (95% CI 0.077–0.256; *p* < 0.001) compared with 0.179 (95% CI 0.095–0.262) in the principal analysis. Substantial heterogeneity persisted (I² = 83.32%; τ² = 0.0119; Q(6) = 40.86, *p* < 0.001) (Fig. 6).

The exclusion of the Michailides et al. cohort did not affect the PSP versus PCT analysis because this study did not report a PCT comparison. Therefore, the pooled PSP versus PCT estimate remained unchanged. Furthermore, because Michailides et al. contributes readmission and therapeutic-escalation outcomes rather than mortality, the seven cohorts remaining after its exclusion from the CRP comparison are the same seven cohorts included in the CRP mortality-only analysis; Figs. 2 and 6 therefore describe numerically equivalent analyses rather than independent corroborating checks. Similarly, all six cohorts contributing to the principal PSP-versus-PCT analysis report mortality, so the PCT mortality-only analysis (Fig. 3) is numerically identical to the principal conservative PCT analysis (Fig. 5). These figures are retained for completeness and clarity of presentation but should not be read as independent replications of the main finding.

### Sensitivity analysis for the assumed within-study correlation

The influence of the assumed correlation between paired AUC estimates within the study was examined by repeating the main conservative analysis using ρ values of 0.30 and 0.80, in addition to the pragmatic value of 0.60 used in the primary analysis. For the comparison of PSP versus CRP, the pooled ΔAUC remained consistent across the different correlation assumptions, ranging from 0.175 (95% CI 0.090–0.261) at ρ = 0.30 to 0.181 (95% CI 0.099–0.263) at ρ = 0.80, with all estimates being statistically significant (*p* < 0.001). In the comparison of PSP versus PCT, the pooled ΔAUC consistently remained at 0.153, with 95% confidence intervals of 0.101–0.206, 0.114–0.193, and 0.124–0.182 for ρ values of 0.30, 0.60, and 0.80, respectively (all *p* < 0.001). These analyses suggest that the direction and approximate magnitude of the pooled effect estimates were preserved across the alternative within-study correlation assumptions. However, heterogeneity estimates, particularly for the PSP versus CRP comparison, varied according to the assumed correlation coefficient (Table 2).

**Table 2:** Sensitivity analysis according to the assumed within-study correlation between paired AUC estimates

Comparator	ρ	k	Pooled ΔAUC	95% CI	*p*	I²	τ²
CRP	0.30	8	0.175	0.090–0.261	< 0.001	70.12%	0.0104
CRP	0.60	8	0.179	0.095–0.262	< 0.001	81.65%	0.0116
CRP	0.80	8	0.181	0.099–0.263	< 0.001	90.05%	0.0124
PCT	0.30	6	0.153	0.101–0.206	< 0.001	0.00%	0.0000
PCT	0.60	6	0.153	0.114–0.193	< 0.001	0.00%	0.0000
PCT	0.80	6	0.153	0.124–0.182	< 0.001	2.75%	0.0000

Pooled differences in area under the receiver operating characteristic curve (ΔAUC) between pancreatic stone protein (PSP) and C-reactive protein (CRP) or procalcitonin (PCT) were estimated in the principal conservative analysis using alternative within-study correlation coefficients (ρ = 0.30, 0.60, and 0.80). Positive ΔAUC values indicate greater prognostic discrimination for PSP. Random-effects models were fitted using restricted maximum likelihood (REML) estimation. The value ρ = 0.60 represents the pragmatic analytical assumption used for the principal analysis, whereas ρ = 0.30 and ρ = 0.80 were examined in sensitivity analyses. k, number of independent cohorts; CI, confidence interval; I², proportion of variability attributable to between-cohort heterogeneity; τ², estimated between-cohort variance

### Risk of bias and applicability

The comparative risk-of-bias assessment revealed significant variation across studies and domains. Patient selection was assessed as low risk in three studies, while four studies were deemed to have unclear risk. The unclear judgments were primarily due to factors such as non-consecutive recruitment, the exclusion of specific patient groups, or incomplete reporting of screened participants. In the index-test domain, four studies were classified as low risk, two as unclear risk, and one as high risk. The concerns in this area included data-driven threshold selection, variability in the availability of biomarker results for treating clinicians, post hoc analyses, and differences in assay procedures. Outcome ascertainment was rated as low risk in five studies and unclear in two. Mortality outcomes were generally well-defined, while greater uncertainty was associated with non-mortality outcomes due to incomplete reporting of the outcome assessment. Regarding flow and timing, two studies were rated as low risk, four as unclear risk, and one as high risk. The high-risk rating was mainly due to incomplete reporting of missing biomarker-specific data, differences in sampling times, incomplete follow-up, or unexplained exclusion of collected samples. Overall, two studies had at least one domain classified as high comparative risk of bias, while four of the remaining five studies had at least one domain rated as unclear. Notably, one study was assessed to be at low risk across all four comparative domains. Concerns about applicability were mainly found in studies involving selected clinical populations, such as patients with postoperative peritonitis and critically ill COVID-19 patients. However, applicability concerns related to prognostic outcomes were considered low across all studies. Detailed assessments for each study are provided in the Supplementary Material.

## Discussion

In this meta-analysis of direct intra-study paired comparisons, PSP demonstrated better prognostic discrimination than CRP and PCT. In the main conservative analysis, which was limited to one comparison per independent cohort and comparator, the pooled change in the area under the curve (ΔAUC) was 0.179 (95% confidence interval [CI] 0.095–0.262) for PSP compared to CRP, and 0.153 (95% CI 0.114–0.193) for PSP compared to PCT. Additional supportive analyses, including eligible paired comparisons, showed effects in a similar direction and of approximately the same magnitude. These findings suggest that the observed comparative discrimination was not solely due to the inclusion of multiple comparisons from the same cohorts. However, the limited number of independent cohorts and clinical heterogeneity necessitate cautious interpretation. It is important to note that there was significant heterogeneity between cohorts in the PSP-versus-CRP comparison (I² = 81.65%), indicating variability in the magnitude of the observed effect. In contrast, no statistical heterogeneity was detected in the PSP-versus-PCT comparison (I² = 0.00%). However, given the limited number of contributing cohorts, this should not be interpreted as evidence of homogeneity of the underlying effects. Thus, while the direction of the comparative effect was generally consistent, especially for PSP compared to CRP, the extent of the prognostic advantage over CRP varied across cohorts and should be interpreted with caution.

This review utilizes direct comparisons of ΔAUC within studies to minimize confounding that may arise from indirect comparisons across different patient populations [7]. Instead of pooling absolute AUC estimates from various studies, which would overlook the paired nature of PSP and comparator measurements obtained from the same participants, we calculated and meta-analyzed the differences in AUC (ΔAUC) within each study-condition unit. This method maintains the paired structure of the data and provides a direct comparison of the prognostic discrimination of PSP versus CRP or PCT within the same patient cohort.Due to the unavailability of empirical covariance information, variance estimation relied on an assumed within-study correlation between paired AUC estimates. Sensitivity analyses, using alternative correlation coefficients (ρ = 0.30 and ρ = 0.80), produced pooled ΔAUC estimates that showed a consistent direction and an approximate magnitude when compared with the pragmatic assumption of ρ = 0.60. However, these analyses do not eliminate the uncertainty stemming from the lack of empirical within-study covariance data. Additionally, heterogeneity estimates, particularly for the PSP versus CRP comparison, varied depending on the assumed correlation coefficient [19, 20]. By restricting the principal conservative analysis to one comparison per independent cohort and comparator, we sought to minimize the potential influence of shared participants, biomarker measurements, and outcome events across multiple study-condition units. The broader supportive analyses yielded results with a similar direction and approximate magnitude, providing complementary evidence that the overall pattern of findings was not materially altered when additional eligible paired comparisons were included. From a clinical perspective, these results suggest that PSP may provide greater prognostic discrimination than traditional biomarkers routinely used in sepsis management. While CRP and PCT remain widely used biomarkers, their limitations for prognostic risk stratification are known. The observed comparative performance of PSP suggests a potential role in early risk stratification at the time of admission or sepsis diagnosis, particularly as part of a multimodal assessment integrating clinical variables and laboratory biomarkers [21]. However, superior AUC performance should not be interpreted as direct evidence of clinical utility. The adoption of a biomarker in routine clinical practice depends on several additional factors, including assay availability, turnaround time, integration into existing laboratory workflows, and cost-effectiveness. Although PSP measurement has become increasingly available in recent years, its accessibility remains more limited than that of CRP and PCT in many healthcare systems. Future prospective studies should determine whether incorporating PSP into existing clinical risk-assessment strategies improves patient classification and meaningfully influences clinical decision-making, resource allocation, or patient-centered outcomes. The substantial heterogeneity observed in the PSP-versus-CRP comparison indicates that the magnitude of the comparative prognostic advantage of PSP varied considerably across cohorts. The included populations encompassed medical and surgical sepsis, intra-abdominal infection, and COVID-19-associated critical illness, including an emergency-department cohort of patients with COVID-19 [15]. The studies also differed with respect to clinical setting (emergency department versus ICU), prognostic outcomes (mortality, multi-organ failure, readmission, and therapeutic escalation), timing of biomarker sampling, assay methodology, and overall methodological quality. These clinical and methodological differences may have contributed to the observed between-cohort heterogeneity. Despite this variability, the direction of the effect generally favoured PSP over CRP across the included comparisons. However, consistency in effect direction should not be interpreted as consistency in effect magnitude, and the pooled PSP-versus-CRP estimate should therefore be interpreted cautiously. In contrast, the PSP-versus-PCT comparison showed little to no between-cohort heterogeneity in the principal conservative analysis, suggesting greater consistency in the magnitude of the comparative effect across the available cohorts. In contrast, no statistical heterogeneity was detected in the PSP-versus-PCT comparison (I² = 0.00%). However, given the limited number of contributing cohorts, this should not be interpreted as evidence of homogeneity of the underlying effects. In contrast, the PSP-versus-PCT comparison showed little to no observed between-cohort heterogeneity in the principal conservative analysis; however, the limited number of contributing cohorts restricts the ability to characterize between-cohort variability.

## Limitations

There are several limitations that need to be acknowledged. First, the number of included studies and independent cohorts was limited, and many of the cohorts had relatively small sample sizes and event counts. There was also substantial clinical and methodological heterogeneity, which reflected differences in patient populations, outcome definitions, biomarker sampling strategies, assay platforms, and methodological quality. The inclusion of multi-organ failure as an additional prognostic outcome represented a deviation from the original protocol, but it was intended to retain important prognostic information from otherwise eligible studies. Despite using random-effects models, substantial heterogeneity remained, especially in the comparison of PSP (Pancreatic Stone Protein) to CRP (C-reactive protein). Therefore, the pooled ΔAUC (change in area under the curve) should be interpreted as an estimate of comparative prognostic discrimination across clinically relevant adverse outcomes, rather than as a specific summary measure of mortality. The analysis relied on aggregate published data, and there was no empirical covariance information for paired AUC estimates available, which necessitated assuming a within-study correlation for variance estimation. Sensitivity analyses that used alternative correlation coefficients preserved the direction and approximate magnitude of the pooled effects; however, they could not eliminate the uncertainty associated with this assumption. Additionally, the heterogeneity estimates for the PSP versus CRP comparison varied depending on the assumed correlation. Michailides et al. [17]. enrolled adults with intra-abdominal infections rather than requiring established sepsis or septic shock at the study entry; only 22 of the 40 participants in this cohort developed sepsis during hospitalization, underscoring its clinical indirectness relative to the review’s target population. Due to the unavailability of subgroup-specific paired AUC data for patients who ultimately developed sepsis, the overall cohort was included. This broadening of the population criterion post-registration may have introduced clinical indirectness and additional heterogeneity. Nevertheless, excluding this cohort did not materially change the direction or approximate magnitude of the pooled effect between PSP and CRP, although substantial heterogeneity still persisted. Furthermore, the broader supportive analyses included multiple paired comparisons from the same cohorts, which may be statistically dependent. If this dependence is not accounted for, it could produce overly precise estimates. Therefore, the analysis that restricted itself to one comparison per independent cohort and each comparator was designated as the principal conservative analysis for inference. Additionally, several studies derived biomarker thresholds from their study populations rather than using externally validated or prespecified cut-offs, which introduces a potential risk of optimism bias. A substantial proportion of the comparative evidence originates from a small number of closely related investigator groups linked to the early development of PSP as a sepsis assay. In particular, several cohorts derive from publications naming an inventor on a University of Zurich-owned PSP patent, and together these cohorts contribute four of the eight independent PSP-versus-CRP comparisons and four of the six PSP-versus-PCT comparisons in the principal analysis. This does not affect the statistical independence of non-overlapping cohorts, but it limits the breadth of independent external replication, especially for the PSP-versus-PCT comparison, whose apparent consistency (I² = 0.00%) should be interpreted in light of this concentration of investigator provenance. Overall, these limitations warrant cautious interpretation of the pooled estimates. The findings should be considered supportive rather than definitive, and further research through larger prospective studies with standardized outcomes, adequately sized cohorts, and harmonized biomarker measurements is needed to better define the comparative prognostic role of PSP.

## Conclusion

In conclusion, the available comparative evidence suggests that PSP may provide greater prognostic discrimination than CRP and PCT. However, the limited number of independent cohorts, the substantial heterogeneity observed in the comparison with CRP, and the methodological limitations of the available evidence warrant cautious interpretation. Further adequately powered prospective studies using standardized outcomes and clinically relevant prediction models are needed to confirm these findings and determine whether differences in discrimination translate into meaningful clinical benefit.

## Supplementary Information

Below is the link to the electronic supplementary material.

Supplementary Material 1

Supplementary Material 2

## Data Availability

Data are included in electronic file in supplementary file to the editor and are available upon request.

## References

[1] Evans L, Rhodes A, Alhazzani W, Antonelli M, Coopersmith CM, French C, Machado FR, McIntyre L, Ostermann M, Prescott HC, Schorr C, Simpson S, Wiersinga WJ, Alshamsi F, Angus DC, Arabi Y, Azevedo L, Beale R, Beilman G, Belley-Cote E, Burry L, Cecconi M, Centofanti J, Coz Yataco A, De Waele J, Dellinger RP, Doi K, Du B, Estenssoro E, Ferrer R, Gomersall C, Hodgson C, Møller MH, Iwashyna T, Jacob S, Kleinpell R, Klompas M, Koh Y, Kumar A, Kwizera A, Lobo S, Masur H, McGloughlin S, Mehta S, Mehta Y, Mer M, Nunnally M, Oczkowski S, Osborn T, Papathanassoglou E, Perner A, Puskarich M, Roberts J, Schweickert W, Seckel M, Sevransky J, Sprung CL, Welte T, Zimmerman J, Levy M (2021) Surviving sepsis campaign: international guidelines for management of sepsis and septic shock 2021. Intensive Care Med 47:1181–1247 10.1007/s00134-021-06506-y PMC8486643 34599691

[2] Llewelyn MJ, Berger M, Gregory M, Ramaiah R, Taylor AL, Curdt I, Lajaunias F, Graf R, Blincko SJ, Drage S, Cohen J (2013) Sepsis biomarkers in unselected patients on admission to intensive or high-dependency care. Crit Care 17:R60 10.1186/cc12588 PMC3672658 23531337

[3] Gkentzi MZO, Gogos D, Akinosoglou C K (2020) Biomarkers in pediatric sepsis: a review of recent literature. Biomark Med 14:895–917 10.2217/bmm-2020-0016 32808806

[4] Ashley BK, Hassan U (2021) Point-of-critical-care diagnostics for sepsis enabled by multiplexed micro and nanosensing technologies. Wiley Interdiscip Rev Nanomed Nanobiotechnol 13:e1701 10.1002/wnan.1701 PMC8447248 33650293

[5] Belletti A, Bonizzoni MA, Labanca R, Osenberg P, Bugo S, Pontillo D, Pieri M, Landoni G, Zangrillo A, Scandroglio AM (2025) Pancreatic Stone Protein as Sepsis Biomarker in Patients Requiring Mechanical Circulatory Support: A Pilot Observational Study. J Cardiothorac Vasc Anesth 39:1229–1235 10.1053/j.jvca.2025.01.037 39971654

[6] Klein HJ, Niggemann P, Buehler PK, Lehner F, Schweizer R, Rittirsch D, Fuchs N, Waldner M, Steiger P, Giovanoli P, Reding T, Graf R, Plock JA (2021) Pancreatic Stone Protein Predicts Sepsis in Severely Burned Patients Irrespective of Trauma Severity: A Monocentric Observational Study. Ann Surg 274:e1179–e1186 10.1097/SLA.0000000000003784 31972652

[7] Fidalgo P, Nora D, Coelho L, Povoa P (2022) Pancreatic Stone Protein: Review of a New Biomarker in Sepsis. J Clin Med 11:1085. 10.3390/jcm11041085 PMC8880320 35207355

[8] García de Guadiana-Romualdo L, Albaladejo-Otón MD, Berger M, Jiménez-Santos E, Jiménez-Sánchez R, Esteban-Torrella P, Rebollo-Acebes S, Hernando-Holgado A, Ortín-Freire A, Trujillo-Santos J (2019) Prognostic performance of pancreatic stone protein in critically ill patients with sepsis. Biomark Med 13:1469–1480 10.2217/bmm-2019-0174 31621373

[9] Gukasjan R, Raptis DA, Schulz HU, Halangk W, Graf R (2013) Pancreatic stone protein predicts outcome in patients with peritonitis in the ICU. Crit Care Med 41:1027–1036 10.1097/CCM.0b013e3182771193 23399938

[10] Bottari G, Paionni E, Fegatelli DA, Murciano M, Rosati F, Ferrigno F, Pisani M, Cristaldi S, Musolino A, Borrelli G, Bochicchio C, Romani L, De Luca M, Agosta M, Lancella L, Villani A, Vestri A, Atti MCD, Perno CF, Porzio O, Raponi M, Cecchetti C (2024) Pancreatic Stone Protein in the Diagnosis of Sepsis in Children Admitted to High-Dependency Care: A Single-Center Prospective Cohort Study. Pediatr Crit Care Med 25:937–946 10.1097/PCC.0000000000003565 39023339

[11] Bottari G, Rosati F, Fegatelli Alunni D, Paionni E, Ferrigno F, Romani L, De Luca M, Lancella L, Porzio O, Bernaschi P, Vestri AR, Ciofi Degli Atti M, Raponi M, Perno CF, Cecchetti C (2025) Pancreatic Stone Protein levels reflect sepsis severity and bloodstream infections in critically ill children: a retrospective cohort analysis. Biomarkers 30:550–559 10.1080/1354750X.2025.2579636 41211644

[12] Page MJ, McKenzie JE, Bossuyt PM, Boutron I, Hoffmann TC, Mulrow CD, Shamseer L, Tetzlaff JM, Akl EA, Brennan SE, Chou R, Glanville J, Grimshaw JM, Hróbjartsson A, Lalu MM, Li T, Loder EW, Mayo-Wilson E, McDonald S, McGuinness LA, Stewart LA, Thomas J, Tricco AC, Welch VA, Whiting P, Moher D (2021) The PRISMA 2020 statement: an updated guideline for reporting systematic reviews. BMJ 372:n71 10.1136/bmj.n71 PMC8005924 33782057

[13] Emir B, Can FE, Kaymaz E, Ozel Z, Efgan MG, Tekindal MA, Elmali F (2026) Sample size and power analysis for ROC AUC differences in diagnostic tests: a methodological evaluation of the Obuchowski-McClish and Hanley-McNeil methods. BMC Med Res Methodol 26:43. 10.1186/s12874-026-02768-6 PMC12924612 41606502

[14] Melegari G, Giuliani E, Di Pietro G, Alberti F, Campitiello M, Bertellini E, Barbieri A (2023) Point-of-care pancreatic stone protein measurement in critically ill COVID-19 patients. BMC Anesthesiol 23:226 10.1186/s12871-023-02187-w PMC10311849 37391718

[15] Van Singer M, Brahier T, Brochu Vez MJ, Gerhard Donnet H, Hugli O, Boillat-Blanco N (2021) Pancreatic stone protein for early mortality prediction in COVID-19 patients. Crit Care 25:267 10.1186/s13054-021-03704-4 PMC8320311 34325711

[16] Que YA, Delodder F, Guessous I, Graf R, Bain M, Calandra T, Liaudet L, Eggimann P (2012) Pancreatic stone protein as an early biomarker predicting mortality in a prospective cohort of patients with sepsis requiring ICU management. Crit Care 16:R114 10.1186/cc11406 PMC3580689 22748193

[17] Michailides C, Lagadinou M, Paraskevas T, Papantoniou K, Kavvousanos M, Vasileiou A, Thomopoulos K, Velissaris D, Marangos M (2023) The Role of the Pancreatic Stone Protein in Predicting Intra-Abdominal Infection-Related Complications: A Prospective Observational Single-Center Cohort Study. Microorganisms 11:2579. 10.3390/microorganisms11102579 PMC10609141 37894237

[18] Que YA, Guessous I, Dupuis-Lozeron E, de Oliveira CRA, Oliveira CF, Graf R, Seematter G, Revelly JP, Pagani JL, Liaudet L, Nobre V, Eggimann P (2015) Prognostication of Mortality in Critically Ill Patients With Severe Infections. Chest 148:674–682 10.1378/chest.15-0123 26065577

[19] Shim SR (2022) Meta-analysis of diagnostic test accuracy studies with multiple thresholds for data integration. Epidemiol Health 44:e2022083 10.4178/epih.e2022083 PMC10106543 36228672

[20] Zhou QM, Zhe L, Brooke RJ, Hudson MM, Yuan Y (2021) A relationship between the incremental values of area under the ROC curve and of area under the precision-recall curve. Diagn Progn Res 5:13 10.1186/s41512-021-00102-w PMC8278775 34261544

[21] Zuercher P, Moser A, Garcia de Guadiana-Romualdo L, Llewelyn MJ, Graf R, Reding T, Eggimann P, Que YA, Prazak J (2023) Discriminative performance of pancreatic stone protein in predicting ICU mortality and infection severity in adult patients with infection: a systematic review and individual patient level meta-analysis. Infection 51:1797–1807 10.1007/s15010-023-02093-w PMC10665254 37707744

