# Supplementary material for: Pancreatic stone protein versus conventional biomarkers for prognostic discrimination in sepsis: a meta-analysis of paired AUC comparisons
Source: Intensive Care Med Exp. 2026 Oct 20;14:140. doi: 10.1186/s40635-026-00990-6 (PMC13683286; doi:10.1186/s40635-026-00990-6)
Supplement: Supplementary file 1 [file 40635_2026_990_MOESM1_ESM.pdf]

NIHR | National Institute for Health and Care Research

PROSPERO
International prospective register of systematic reviews

Pancreatic Stone Protein for Prognostic Prediction in Sepsis and Septic Shock: A Systematic Review and Meta-Analysis Comparing PSP with Procalcitonin and C-Reactive Protein.

Gabriele Melegari, Valerio Valente, Federica Arturi, Ilaria Pacini, Eros Pillia, Alessandro Belletti, Andrea Tognù, Elisabetta Bertellini, Alberto Barbieri

Citation

Gabriele Melegari, Valerio Valente, Federica Arturi, Ilaria Pacini, Eros Pillia, Alessandro Belletti, Andrea Tognù, Elisabetta Bertellini, Alberto Barbieri. Pancreatic Stone Protein for Prognostic Prediction in Sepsis and Septic Shock: A Systematic Review and Meta-Analysis Comparing PSP with Procalcitonin and C-Reactive Protein.. PROSPERO 2026 CRD420261340301. Available from https://www.crd.york.ac.uk/PROSPERO/view/CRD420261340301.

REVIEW TITLE AND BASIC DETAILS

Review title

Pancreatic Stone Protein for Prognostic Prediction in Sepsis and Septic Shock: A Systematic Review and Meta-Analysis Comparing PSP with Procalcitonin and C-Reactive Protein.

Condition or domain being studied

Population (P)

Sepsis

Septic shock

Critical illness

Intensive care unit

Intervention / Index test (I)

Pancreatic stone protein

Biomarkers

Comparator (C)

C-reactive protein

Procalcitonin

Outcome (O)

Mortality

Prognosis

Disease severity

Rationale for the review

the aim of this study was to perform a meta-analysis of direct intra-study comparisons to determine whether Pancreatic Stone Protein provides superior prognostic discrimination compared with C-reactive protein and procalcitonin for predicting critical outcomes in patients with sepsis and septic shock. Critical outcomes were defined a priori as mortality (in-hospital, 28-day, or longer-term), ICU readmission, or need for therapeutic escalation. To date, no quantitative synthesis has specifically pooled within-study ΔAUC values accounting for intra-sample correlation. The primary effect measure was the difference in area under the receiver operating characteristic curve (ΔAUC). This meta-analysis focuses on within-study paired comparisons of AUCs to directly assess the incremental prognostic value of PSP over established biomarkers

Review objectives

This systematic review and meta-analysis aims to evaluate the prognostic performance of pancreatic stone protein (PSP) compared with conventional biomarkers (C-reactive protein and procalcitonin) in adult patients with sepsis or septic shock. The review will synthesize evidence from studies reporting paired intra-study comparisons of biomarker performance to determine whether PSP provides superior prognostic discrimination for clinically relevant outcomes such as mortality, ICU readmission, or need for therapeutic escalation.

Keywords

Pancreatic stone protein; Procalcitonin; C-reactive protein CRP

Country

Italy

ELIGIBILITY CRITERIA

Population

Included
Adult patients with sepsis or septic shock managed in acute care settings (intensive care units, emergency departments, or hospital wards) in whom pancreatic stone protein (PSP) and comparator biomarkers (C-reactive protein and/or procalcitonin) were measured for prognostic assessment of critical clinical outcomes, including mortality, ICU readmission, or need for therapeutic escalation.

Only studies involving human adult populations will be included.

Excluded
Studies will be excluded if they meet any of the following criteria:

Studies including pediatric populations only.

Studies conducted in non-infectious or non-sepsis populations.

Studies evaluating diagnostic performance only, without reporting prognostic outcomes.

Studies not reporting AUC (area under the receiver operating characteristic curve) values for pancreatic stone protein and at least one comparator biomarker (C-reactive protein or procalcitonin), or not allowing extraction or derivation of 95% confidence intervals.

Studies conducted in non-clinical or experimental settings (e.g., animal studies, laboratory studies).

Case reports, editorials, letters, narrative reviews, and conference abstracts without sufficient data.

Duplicate publications or overlapping datasets, in which case the most complete dataset will be included.

Intervention(s) or exposure(s)

Included
Studies will be included when PSP is measured in blood samples at hospital admission, ICU admission, or during the early phase of sepsis, and when its prognostic performance is evaluated in comparison with conventional biomarkers such as C-reactive protein (CRP) and procalcitonin (PCT).

Excluded
Intervention(s) or exposure(s) exclusion criteria

Studies will be excluded if pancreatic stone protein (PSP) is not measured in blood samples or if PSP is not evaluated as a prognostic biomarker for clinical outcomes in patients with sepsis or

septic shock.

Studies will also be excluded if:

PSP is evaluated only for diagnostic purposes and not for prognostic assessment.

PSP measurements are not reported alongside at least one comparator biomarker (C-reactive protein or procalcitonin) in the same patient population.

The study does not provide sufficient data to extract or derive AUC values and corresponding confidence intervals.

Comparator(s) or control(s)

Included
PICO tags selected: Usual Care

Study design

Only nonrandomized study types will be included.

Included
Prospective or retrospective observational studies, including cohort and case–control studies, evaluating the prognostic performance of pancreatic stone protein (PSP) in adult patients with sepsis or septic shock. Studies must report AUC values for PSP and at least one comparator biomarker (CRP or procalcitonin) measured in the same population.

Excluded
Case reports, case series without comparator biomarkers, narrative reviews, editorials, letters, conference abstracts without sufficient data, and experimental or animal studies will be excluded.

Studies focusing exclusively on diagnostic performance without reporting prognostic outcomes, as well as studies conducted exclusively in pediatric populations, will also be excluded.

Context

Acute care settings including intensive care units (ICU), emergency departments, and hospital wards where patients with sepsis or septic shock are managed.

The review focuses on studies evaluating pancreatic stone protein and comparator biomarkers in critically ill or acutely ill patients with infection in hospital settings.

TIMELINE OF THE REVIEW

Date of first submission to PROSPERO

19 March 2026

Review timeline

Start date: 13 March 2026. End date: 1 May 2026.

Date of registration in PROSPERO

24 March 2026

AVAILABILITY OF FULL PROTOCOL

Availability of full protocol

A full protocol has been written but is not available because:

No full protocol has been published. The methodological details are provided within this PROSPERO registration.

SEARCHING AND SCREENING

Search for unpublished studies

Only published studies will be sought.

Main bibliographic databases that will be searched

The main databases to be searched are *Embase - Embase via Ovid*, *Embase.com*, *MEDLINE*, *PubMed* and *Scopus*.

Search language restrictions

There are no language restrictions.

Search date restrictions

There are no search date restrictions.

Other methods of identifying studies

Other studies will be identified by: *searching trial or study registers*.

Link to search strategy

A full search strategy is not available.

Selection process

Studies will be screened independently by at least two people (or person/machine combination) with a process to resolve differences.

Other relevant information about searching and screening

Two reviewers will independently screen titles and abstracts identified through database searches. Full-text articles will be assessed for eligibility according to predefined inclusion and exclusion criteria. Any disagreements will be resolved by discussion and consensus, with involvement of a third reviewer if necessary. Reference lists of included studies and relevant reviews will be screened to identify additional eligible studies. Duplicate records will be removed before screening.

DATA COLLECTION PROCESS

Data extraction from published articles and reports

Data will be extracted by one person (or a machine) and checked by at least one other person (or machine).

Authors will not be contacted for further information.

Study risk of bias or quality assessment

Data will be assessed independently by at least two people (or person/machine combination) with a process to resolve differences.

Additional information will be sought from study investigators if required information is unclear or unavailable in the study publications/reports.

Reporting bias assessment

Risk of bias due to missing results will not be assessed

Certainty assessment

Certainty of findings will not be assessed

OUTCOMES TO BE ANALYSED

Main outcomes

Main outcomes

The main outcome is the prognostic performance of pancreatic stone protein compared with conventional biomarkers (C-reactive protein and procalcitonin) for predicting critical clinical outcomes in patients with sepsis or septic shock.

Prognostic discrimination will be assessed using the area under the receiver operating characteristic curve (AUC). The primary effect measure will be the difference in AUC (ΔAUC) between pancreatic stone protein and comparator biomarkers.

Critical outcomes include:

mortality (in-hospital or short-term mortality)

ICU readmission

need for therapeutic escalation

Additional outcomes

Additional outcomes include pooled analyses by comparator biomarker (CRP and PCT), assessment of between-study heterogeneity, sensitivity analyses under different statistical assumptions, and evaluation of small-study effects.

PLANNED DATA SYNTHESIS

Strategy for data synthesis

Yes. A quantitative meta-analysis will be performed. The primary effect size will be the difference in area under the receiver operating characteristic curve (ΔAUC) between pancreatic stone protein and comparator biomarkers (CRP or procalcitonin) measured within the same

study population. Pooled estimates will be calculated using random-effects models to account for between-study heterogeneity.

CURRENT REVIEW STAGE

Stage of the review at this submission

Review stage	Started	Completed
Pilot work	✓	
Formal searching/study identification	✓	
Screening search results against inclusion criteria	✓	
Data extraction or receipt of IPD		
Risk of bias/quality assessment		
Data synthesis		

Review status

The review is currently planned or ongoing.

Publication of review results

Results of the review will be published in English.

REVIEW AFFILIATION, FUNDING AND PEER REVIEW

Review team members

Dr Gabriele Melegari (review guarantor and contact) ORCID: 0000-0003-4512-4835. Azienda Ospedaliero-Universitaria di Modena. Italy.

No conflict of interest declared.

Valerio Valente. Università di Modena e Reggio Emilia. Italy.

No conflict of interest declared.

Dr Federica Arturi. University of Modena and Reggio Emilia. Italy.

No conflict of interest declared.

Ilaria Pacini. Ospedale Rizzoli. Italy.

No conflict of interest declared.

Dr Eros Pillia. Ospedale Broztu Cagliari. Italy.

No conflict of interest declared.

Dr Alessandro Belletti. Ospedale Vita e Salute San Raffaele Milano. Italy.

No conflict of interest declared.

Dr Andrea Tognù. Gaetano Pini, Milano. Italy.

No conflict of interest declared.

Dr Elisabetta Bertellini. Università di Modena e Reggio Emilia. Italy.

No conflict of interest declared.

Professor Alberto Barbieri. Università degli Studi di Modena e Reggio Emilia. Italy.

No conflict of interest declared.

Named contact

Dr Gabriele Melegari (gabriele.melegari@unimore.it). ORCID: 0000-0003-4512-4835. Azienda Ospedaliero-Universitaria di Modena. Italy.

Review affiliation

Università degli Studi di Modena e Reggio Emilia

Funding source

Review has no specific/external funding but is supported by guarantor/review team (non-commercial) institutions.

Peer review

There has been no peer review of this planned review.

ADDITIONAL INFORMATION

Review conflict of interest

Declared individual interests are recorded under team member details.. No additional interests are recorded for this review.

Medical Subject Headings

Adult; Biomarkers; C-Reactive Protein; Classification; Hospitals; Humans; Intensive Care Units; Lithostathine; Patient Readmission; Procalcitonin; Prognosis; Shock, Septic

SIMILAR REVIEWS

Check for similar records already in PROSPERO

PROSPERO identified a number of existing PROSPERO records that were similar to this one (last check made on 19 March 2026). These are shown below along with the reasons given by that the review team for the reviews being different and/or proceeding.

- Diagnostic Accuracy of Pancreatic Stone Protein (PSP) for Sepsis for Patients Presenting to the Emergency Department: A Systematic Review and Meta-analysis Protocol [published 22 January 2026] [CRD420261288498]. The review was judged **not to be similar**
- Association between C-Reactive Protein to Albumin Ratio and Mortality: A Systematic Review and Meta-analysis. [published 7 November 2025] [CRD420251185977]. The review was judged **not to be similar**
- Performance of Pancreatic Stone Protein for Severity Stratification and Prognostication of Outcome in Hospitalized Adults with Infection: A Systematic Review and Individual Patient Level Meta-analysis [published 25 March 2022] [CRD42022308207]. The review was acknowledged as **similar** to the current review and the authors chose to abandon it.
- Diagnostic and prognostic performance of C-reactive protein and procalcitonin in sepsis among patients with chronic kidney disease: A systematic review and meta-analysis [published 17 March 2026] [CRD420261342861]. The review was judged **not to be similar**

- Prognostic Significance of Serum C-Reactive Protein in Oral Potentially Malignant Disorders: A Systematic Review [published 19 February 2026] [CRD420261320628]. The review was judged **not to be similar**
- C-reactive protein as the prognostic factors for tuberculosis outcome: Systematic review and meta-analysis [published 14 July 2025] [CRD420251101984]. The review was judged **not to be similar**
- Diagnostic value of serum pancreatic stone protein for sepsis: A meta-analysis [published 8 March 2022] [CRD42022308446]. The review was judged **not to be similar**
- Prognostic Value of Admission CRP for Adverse Outcomes in Severe Odontogenic and Mixed Head and Neck Infections: Systematic Review and Single-Arm Meta-analysis. [published 30 January 2026] [CRD420261288715]. The review was judged **not to be similar**
- Prognostic Accuracy of C-Reactive Protein for Identifying Severe Odontogenic Abscesses at Hospital Admission: A Systematic Review and Meta-Analysis [published 29 June 2025] [CRD420251083571]. The review was judged **not to be similar**
- The C-reactive protein as influence of prognostic factor in patients with acute ischemic stroke: a systematic review and meta-analysis [published 15 January 2015] [CRD42015016001]. The review was judged **not to be similar**

PROSPERO version history

- Version 1.0, published 24 Mar 2026

Disclaimer

The content of this record displays the information provided by the review team. PROSPERO does not peer review registration records or endorse their content.

PROSPERO accepts and posts the information provided in good faith; responsibility for record content rests with the review team. The guarantor for this record has affirmed that the information provided is truthful and that they understand that deliberate provision of inaccurate information may be construed as scientific misconduct.

PROSPERO does not accept any liability for the content provided in this record or for its use. Readers use the information provided in this record at their own risk.

Any enquiries about the record should be referred to the named review contact
