# Supplementary material for: Pancreatic stone protein versus conventional biomarkers for prognostic discrimination in sepsis: a meta-analysis of paired AUC comparisons
Source: Intensive Care Med Exp. 2026 Oct 20;14:140. doi: 10.1186/s40635-026-00990-6 (PMC13683286; doi:10.1186/s40635-026-00990-6)
Supplement: Supplementary file 2 [file 40635_2026_990_MOESM2_ESM.docx]

**Supplementary material**

Supplementary Search Strategies
Database-specific search strategies for PubMed, EMBASE, and Scopus are reported below. Clinical eligibility criteria related to sepsis, septic shock, and acute infection-related conditions were applied during study screening, as described in the main manuscript**.**

**Pubmed®**

- ("pancreatic stone protein"[All Fields]
- OR "pancreatic stone protein/regenerating protein"[All Fields]
- OR "pancreatic stone protein/reg"[All Fields]
- OR "PSP/reg"[All Fields]
- OR "REG1A"[All Fields]
- OR "REG1 alpha"[All Fields]
- OR "REG I alpha"[All Fields]
- OR "lithostathine"[All Fields]
- OR "lithostatin"[All Fields])

**Embase®**

- 'pancreatic stone protein':ti,ab,kw
- OR 'pancreatic stone protein/regenerating protein':ti,ab,kw
- OR 'pancreatic stone protein/reg':ti,ab,kw
- OR 'psp/reg':ti,ab,kw
- OR 'reg1a':ti,ab,kw
- OR 'reg1 alpha':ti,ab,kw
- OR 'reg i alpha':ti,ab,kw
- OR 'lithostathine':ti,ab,kw
- OR 'lithostatin':ti,ab,kw

**Scopus®**

- TITLE-ABS-KEY(
- "pancreatic stone protein"
- OR "pancreatic stone protein/regenerating protein"
- OR "pancreatic stone protein/reg"
- OR "PSP/reg"
- OR "REG1A"
- OR "REG1 alpha"
- OR "REG I alpha"
- OR "lithostathine"
- OR "lithostatin")

Funding and competing interests of included studies

Funding and competing interests: Funding sources and declared competing interests were reviewed for the included studies. Of particular relevance to the interpretation of the present meta-analysis, Que et al. (2012), Que et al. (2015), and Gukasjan et al. (2013) share an investigator who was identified in the competing-interests statement of Que et al. (2012) as an inventor on a University of Zurich-owned patent relating to PSP as a sepsis assay. These publications contribute four of the eight independent cohorts included in the principal PSP-versus-CRP analysis and four of the six cohorts included in the principal PSP-versus-PCT analysis. This concentration of investigator provenance does not affect the statistical independence of non-overlapping cohorts but limits the breadth of independent external replication

**Review specific decision rules**

A domain was classified as low risk of bias when the available information was sufficient to exclude a relevant source of comparative bias; high risk of bias when one or more features of study design, conduct, analysis, or reporting provided a plausible mechanism by which the relative performance of the compared biomarkers could be biased; and unclear risk of bias when reporting was insufficient to support either a low- or high-risk judgment.

Because the objective of the meta-analysis was the direct comparison of prognostic discrimination between biomarkers measured within the same participants, the assessment focused primarily on sources of bias that could affect the relative comparison between PSP and CRP and/or PCT. Factors potentially affecting the absolute prognostic performance of all biomarkers similarly were therefore not automatically considered sources of comparative bias unless they could plausibly influence the relative comparison.

For patient selection, judgments considered whether recruitment was consecutive or random, whether a case-control design was avoided, whether exclusions were appropriate, and whether the compared biomarkers were evaluated in the same participants. Particular attention was given to selection mechanisms that could alter the clinical or prognostic spectrum of the study population.

For the index-test domain, judgments considered whether PSP and comparator biomarkers were obtained at comparable time points, whether measurement and interpretation were performed independently, whether prespecified or data-derived thresholds were used, whether assay procedures differed in ways that could favour one biomarker, and whether biomarker results were available to treating clinicians. Data-driven threshold optimisation was considered a potential source of bias in absolute prognostic performance; it contributed to comparative risk of bias when it could plausibly affect the relative comparison between biomarkers.

For outcome ascertainment, the diagnostic reference-standard construct of QUADAS-2/QUADAS-C was adapted to the prognostic setting. Judgments considered whether the prognostic outcome was clearly and objectively defined, whether ascertainment was independent of the biomarker results, whether any biomarker formed part of the outcome definition, and whether the same outcome definition and follow-up period were applied to all compared biomarkers. Mortality was generally considered an objective outcome, whereas outcomes involving clinical judgement, such as therapeutic escalation or multi-organ failure, required additional consideration of definition and assessor blinding.

For flow and timing, judgments considered whether the biomarkers were measured at clinically comparable time points, whether measurement preceded the prognostic outcome, whether the same participants contributed to each paired biomarker comparison, and whether missing biomarker or outcome data were comparable across index tests. Unexplained exclusions, differential biomarker availability, or incomplete reporting of biomarker-specific denominators were considered potential sources of comparative bias.

When a study contributed more than one eligible prognostic outcome, the study-level assessment considered all outcomes contributing to the meta-analysis. Where domain-specific risk differed across outcomes, the more conservative judgment was retained for the study-level summary. Similarly, when reporting was insufficient to determine whether an apparent methodological limitation affected the compared biomarkers equally, an unclear rather than a low-risk judgment was assigned

**Applicability assessment**

Applicability concerns were evaluated separately from risk of bias and were classified as low, high, or unclear. Population applicability considered the extent to which enrolled participants represented the target population of adults with sepsis, septic shock, or clinically relevant acute infection-related conditions. Biomarker applicability considered whether the assays and measurement procedures corresponded to the prognostic biomarker comparison addressed by the meta-analysis. Outcome applicability considered whether the evaluated endpoints represented clinically relevant adverse outcomes consistent with the review question.

Importantly, applicability concerns were not automatically interpreted as risk of comparative bias. A study conducted in a clinically specific population could therefore have limited applicability to the broader sepsis population while still providing an internally valid paired comparison between PSP and the comparator biomarker.

**García de Guadiana-Romualdo et al. (2019)**

The study prospectively enrolled 122 consecutive ICU patients with sepsis or septic shock. PSP, CRP, and PCT were assessed within the same cohort and compared against 28-day mortality. Eleven of 133 screened patients were excluded, including six because of limitation of therapeutic effort. Baseline biomarkers were obtained within six hours of sepsis diagnosis; CRP and PCT were measured in the central laboratory, whereas PSP was subsequently measured from stored serum.

| Domain | Key signalling questions | Judgment | Rationale |
| --- | --- | --- | --- |
| Patient selection | Consecutive enrolment? Yes; case-control avoided? Yes; inappropriate exclusions avoided? No/Unclear; fully paired comparison? Yes | Unclear | Exclusion of patients with limitation of therapeutic effort may have altered the prognostic spectrum. However, the same selection criteria applied to all biomarkers, limiting the likelihood of differential selection bias. |
| Index tests | Comparable sampling? Yes; independent measurement? Yes; prespecified thresholds? No; differential conduct between tests? No clear evidence | Low | All biomarkers originated from the same baseline clinical assessment. Although optimal cut-offs were data-derived, the present meta-analysis used threshold-independent AUC estimates, and no clear mechanism favouring one biomarker in the paired AUC comparison was identified. |
| Outcome ascertainment | Appropriate outcome? Yes; independent of biomarker results? Yes; same outcome for all tests? Yes | Low | Twenty-eight-day all-cause mortality was an objective outcome, defined identically for all biomarker comparisons and independent of biomarker values. |
| Flow and timing | Appropriate timing? Yes; same participants/outcome? Yes; differential missingness? No evidence | Low | Baseline biomarkers were obtained before outcome ascertainment from the same clinical sampling episode. Attrition before day 2 did not affect the baseline comparison used in the principal analysis. |

Applicability concerns: Low for population, index tests, and outcome.

Overall rationale: The main comparative concern was patient selection, because exclusion of patients with limitation of therapeutic effort could have modified the prognostic spectrum. The fully paired design, common baseline sampling, and objective mortality endpoint otherwise limited the potential for differential bias between PSP and the comparator biomarkers.

**Gukasjan et al. (2013)**

This prospective cohort included **91 consecutive postoperative patients with proven secondary peritonitis**, admitted to a medical-surgical ICU. Blood samples were collected within 3 hours of ICU admission. Patients were excluded if blood sampling was not obtained within that window or if they were transferred from another hospital. PSP, CRP and PCT were measured in the same cohort, but clinicians were blinded to PSP while CRP and PCT were available as routine clinical tests.

| Domain | Key signalling questions | Judgment | Rationale |
| --- | --- | --- | --- |
| Patient selection | Consecutive enrolment? Yes; case-control avoided? Yes; inappropriate exclusions avoided? Yes; fully paired comparison? Yes | Low | Consecutive recruitment and clearly reported eligibility criteria support a low risk of comparative selection bias. PSP, CRP and PCT were assessed in the same participants. |
| Index tests | Comparable sampling? Yes; independent measurement? Yes; prespecified thresholds? No; differential clinical availability? Yes | High | PSP was unavailable to clinicians, whereas CRP and PCT were available during routine care. The authors explicitly acknowledged that this asymmetry could have influenced management and outcomes. Cut-offs were also derived from ROC analyses using the Youden index. |
| Outcome ascertainment | Clearly defined outcome? Partly; independent of biomarkers? Unclear; same outcomes for all tests? Yes | Unclear | ICU mortality was objective, but the study also contributed multi-organ failure, for which operational definition and adjudication were less clearly reported. Awareness of CRP/PCT but not PSP could also have influenced management-related outcomes. |
| Flow and timing | Appropriate timing? Yes; same participants? Yes; differential missingness adequately reported? Unclear | Unclear | Biomarkers were sampled within the same 3-hour admission window, but detailed reporting of screening exclusions and biomarker-specific missing or invalid measurements was insufficient to support a low-risk judgment. |

Applicability concerns: High for population, because the cohort consisted specifically of postoperative patients with secondary peritonitis rather than a broad sepsis population; low for index tests and outcome.

Overall rationale: The paired and consecutive design limits comparative selection bias. The main concern is the index-test domain, because PSP results were blinded while CRP and PCT were clinically available, creating a plausible mechanism for differential influence on patient management and subsequent outcomes. Outcome ascertainment and flow/timing were judged unclear because of incomplete reporting, particularly for multi-organ failure and biomarker-level completeness. The study nevertheless provides a direct within-cohort comparison of PSP with CRP and PCT.

**Melegari et al. (2023)**

This prospective proof-of-concept study enrolled 21 mechanically ventilated adults admitted to a COVID-19 ICU. PSP, CRP, and PCT were measured at ICU admission (T0) and serially thereafter, and 90-day mortality was obtained from an electronic register. Of 109 patients admitted during the study period, 21 met the eligibility criteria and were enrolled.

| Domain | Key signalling questions | Judgment | Rationale |
| --- | --- | --- | --- |
| Patient selection | Consecutive/random enrolment? Unclear; case-control avoided? Yes; inappropriate exclusions avoided? Unclear; fully paired comparison? Yes | Unclear | Patients were prospectively screened using predefined criteria, but only 21 of 109 ICU admissions were enrolled and consecutive recruitment was not explicitly reported. The restrictive eligibility criteria selected a highly specific critically ill COVID-19 population, although the same selection process applied to all biomarkers. |
| Index tests | Comparable sampling? Yes; independent/objective measurement? Yes; prespecified thresholds? Unclear; differential conduct between tests? No clear evidence | Low | PSP, CRP, and PCT were measured at the same predefined time points. PSP was measured by point-of-care fluorescent immunoassay and CRP/PCT by routine laboratory testing. The comparative analysis was based on AUCs, and no clear differential mechanism favouring one biomarker in the paired comparison was identified. |
| Outcome ascertainment | Clearly defined outcome? Yes; independent of biomarker results? Yes; same outcome for all tests? Yes | Low | Ninety-day mortality was an objective endpoint obtained from an electronic register and was applied identically to PSP, CRP, and PCT. |
| Flow and timing | Appropriate timing? Yes; complete paired measurements? No; missing data adequately explained? Partly | High | Although sampling times were predefined, 138 samples were collected, 84 were included in the analysis, and PSP could not be measured in two additional samples, leaving 82 PSP measurements. The substantial reduction in analysed samples and differential PSP availability creates a plausible risk of bias in the paired comparison. |

Applicability concerns: High for population, because the study was restricted to mechanically ventilated patients with critical COVID-19 rather than a broader sepsis population; low for index tests and outcome.

Overall rationale: The study provided direct comparisons of PSP with CRP and PCT at predefined time points and used an objective mortality endpoint. Comparative risk of bias was considered unclear for patient selection because consecutive enrolment was not explicitly documented. The principal concern was flow and timing, owing to the substantial reduction from collected to analysed samples and the additional absence of PSP measurements. The highly selected COVID-19 ICU population also limits applicability to the broader sepsis population.

**Michailides et al. (2023)**

This prospective single-centre cohort included 40 consecutive adults admitted with intra-abdominal infection. PSP was measured within the first 24 hours using the abioSCOPE point-of-care platform, and prognostic outcomes included treatment escalation and 30-day readmission. The study explicitly used consecutive sampling and avoided a case-control design.

| Domain | Key signalling questions | Judgment | Rationale |
| --- | --- | --- | --- |
| Patient selection | Consecutive enrolment? Yes; case-control avoided? Yes; inappropriate exclusions avoided? Yes; fully paired comparison? Yes | Low | Consecutive recruitment and predefined eligibility criteria support a low risk of comparative selection bias. PSP and CRP were assessed within the same cohort. |
| Index tests | Comparable sampling? Yes; independent analysis? Yes; prespecified thresholds? No; differential conduct between tests? No clear evidence | Low | PSP and CRP were measured from the same clinical assessment. Cut-offs were derived using the Youden index and no threshold was prespecified, but the meta-analysis used AUC estimates rather than threshold-dependent measures. The data analyst was blinded to outcome coding, and no clear mechanism favouring one biomarker in the comparative AUC assessment was identified. |
| Outcome ascertainment | Clearly defined outcomes? Yes; independent of biomarker results? Unclear; same outcomes for both tests? Yes | Unclear | Readmission was relatively objective, whereas treatment escalation depended partly on clinical course and management decisions. Although outcomes were prospectively defined, blinding of treating clinicians to biomarker results was not clearly reported. |
| Flow and timing | Appropriate timing? Yes; same participants? Mostly; complete outcome data? No for readmission | Unclear | Biomarkers were obtained at baseline before subsequent outcomes. Treatment-escalation data were available for the full cohort, whereas readmission data were available for 36 of 40 patients. The limited missing follow-up prevents a confident low-risk judgment. |

Applicability concerns: High for population, because participants were enrolled on the basis of intra-abdominal infection and did not necessarily have established sepsis or septic shock at baseline; low for index tests and outcome. This distinction is particularly important here because it reflects the post-registration broadening of the population criterion rather than comparative bias within the study itself. The source paper reports that only 22 of the 40 participants developed sepsis during hospitalization.

Overall rationale: The study used consecutive recruitment and a fully paired comparison, supporting low comparative risk in patient selection. The main uncertainties concern outcome ascertainment and flow: treatment escalation includes an element of clinical judgment, clinician blinding was not clearly documented, and readmission follow-up was incomplete in four participants. The population itself is clinically narrower and more indirect relative to the broader sepsis population of the meta-analysis.

**Que et al. (2012)**

This prospective single-centre cohort evaluated adults with severe sepsis or septic shock within 24 hours of admission to a mixed medical-surgical ICU. Recruitment was prospective but not strictly consecutive because of organisational constraints. Of 177 patients screened, 25 declined consent and 18 died before consent could be obtained; 107 patients were ultimately included.

| Domain | Key signalling questions | Judgment | Rationale |
| --- | --- | --- | --- |
| Patient selection | Consecutive enrolment? No; case-control avoided? Yes; inappropriate exclusions avoided? Unclear; fully paired comparison? Yes | Unclear | Recruitment was not strictly consecutive, and 18 screened patients died before consent could be obtained. This may have preferentially excluded patients at highest mortality risk. However, the same cohort was used for all biomarker comparisons. |
| Index tests | Comparable sampling? Yes; independent measurement? Unclear; prespecified thresholds? Not relevant to AUC comparison; differential conduct between tests? No clear evidence | Unclear | PSP, CRP, and PCT were measured within 24 hours of ICU admission and compared using AUCs. However, reporting was insufficient to establish blinding or completely exclude differences in measurement/interpretation that might affect the comparative assessment. |
| Outcome ascertainment | Clearly defined outcome? Yes; independent of biomarkers? Yes; same outcome for all tests? Yes | Low | In-hospital mortality was an objective outcome and was used identically for PSP, CRP, and PCT. ROC curves for all biomarkers used mortality as the same dependent variable. |
| Flow and timing | Appropriate timing? Yes; same participants? Yes; complete paired data clearly documented? Unclear | Unclear | Biomarkers were obtained within the same early ICU period and preceded mortality. However, biomarker-specific completeness and the exact denominators contributing to each paired ROC analysis were not reported in sufficient detail to confidently exclude differential missingness. |

Applicability concerns: Low for population, index tests, and outcome. The cohort directly comprised adults with severe sepsis or septic shock admitted to ICU, and in-hospital mortality is directly relevant to the review question. The study included 33 patients with severe sepsis and 74 with septic shock.

Overall rationale: The principal concern relates to patient selection because recruitment was not strictly consecutive and patients who died before consent could be obtained were excluded, potentially modifying the mortality-risk spectrum. Outcome ascertainment was robust because mortality was objective and identical across biomarkers. Incomplete reporting of blinding and biomarker-specific analytical completeness prevents confident low-risk judgments for the index-test and flow/timing domains.

**Que et al. (2015)**

This prospective study included two independent cohorts of adults with severe sepsis or septic shock: a derivation cohort in Switzerland (n = 158) and an external validation cohort in Brazil (n = 91). Biomarkers, including CRP, PCT, and PSP, were obtained at the time of sepsis diagnosis and evaluated against in-hospital mortality.

| Domain | Key signalling questions | Judgment | Rationale |
| --- | --- | --- | --- |
| Patient selection | Consecutive enrolment? No/Unclear; case-control avoided? Yes; inappropriate exclusions avoided? Unclear; fully paired comparison? Yes | Unclear | Recruitment was prospective, but the authors explicitly state that enrolment in the derivation cohort could not be strictly consecutive because of organizational constraints. The same participants contributed to the biomarker comparisons. |
| Index tests | Comparable sampling? Yes; independent measurement? Unclear; prespecified thresholds? Not relevant to AUC comparison; differential conduct between tests? No clear evidence | Unclear | CRP, PCT, and PSP were assessed at the time of sepsis diagnosis within the same cohorts. However, reporting on blinding and biomarker-specific analytical procedures was insufficient to confidently exclude differential measurement or interpretation bias. |
| Outcome ascertainment | Clearly defined outcome? Yes; independent of biomarkers? Yes; same outcome for all tests? Yes | Low | In-hospital mortality was an objective outcome and was assessed identically for PSP, CRP, and PCT in both cohorts. |
| Flow and timing | Appropriate timing? Yes; same participants? Yes; biomarker-specific completeness adequately reported? Unclear | Unclear | Biomarkers were obtained at diagnosis and before the mortality outcome, but detailed biomarker-specific denominators and missing-data information were not sufficiently reported to support a low-risk judgment. |

Applicability concerns**:** Low for population, index tests, and outcome. Both cohorts directly included adults with severe sepsis or septic shock requiring ICU care.

The two cohorts differed substantially in disease severity and mortality: hospital mortality was 27% in the derivation cohort and 43% in the validation cohort. The authors also acknowledged uncertainty in the timing between infection onset and biomarker sampling, particularly between the Swiss and Brazilian settings. These differences affect clinical heterogeneity and generalizability but do not, by themselves, demonstrate comparative bias between biomarkers.

Overall rationale: The study provides fully paired biomarker comparisons in two independent prospective cohorts and uses an objective mortality endpoint. The main concerns are non-strictly consecutive recruitment in the derivation cohort and incomplete reporting of blinding, assay completeness, and biomarker-specific missing data. These limitations support Unclear, rather than High, comparative risk of bias in patient selection, index tests, and flow/timing.

**Van Singer et al. (2021)**

This prospective cohort included 173 consecutive adults presenting to the emergency department with RT-PCR-confirmed COVID-19. PSP and CRP were evaluated at ED presentation for prediction of 7-day mortality. PSP was measured retrospectively from stored plasma, whereas CRP was measured as part of routine admission testing.

| Domain | Key signalling questions | Judgment | Rationale |
| --- | --- | --- | --- |
| Patient selection | Consecutive enrolment? Yes; case-control avoided? Yes; inappropriate exclusions avoided? Yes; fully paired comparison? Yes | Low | Consecutive prospective recruitment was explicitly reported, and PSP and CRP were compared within the same participant cohort. |
| Index tests | Comparable sampling? Yes; independent measurement? Yes/likely; prespecified thresholds? No; differential conduct between tests? No clear evidence | Low | PSP was measured retrospectively from stored ED plasma, whereas CRP was measured routinely at admission. Both reflected the same clinical presentation. Optimal cut-offs were data-derived using the Youden index, but the meta-analysis used threshold-independent AUROCs. |
| Outcome ascertainment | Clearly defined outcome? Yes; independent of PSP? Yes; same outcome for both tests? Yes | Low | Seven-day mortality was an objective outcome and was applied identically to PSP and CRP. Twelve of 173 patients died within 7 days. |
| Flow and timing | Appropriate timing? Yes; same participants? Yes; differential missingness reported? No clear evidence of relevant missingness | Low | Both biomarkers corresponded to ED presentation and preceded the mortality endpoint. No relevant differential loss from the paired mortality analysis was reported. |

Applicability concerns: High for population, because this was an emergency-department cohort with COVID-19 rather than a cohort of patients with established sepsis or septic shock. Applicability was low for the index tests and outcome. The population difference is therefore principally an issue of indirectness/generalizability rather than internal comparative bias.

Overall rationale: The study had a strong comparative structure, with consecutive prospective recruitment, paired biomarker assessment at presentation, and an objective short-term mortality endpoint. Although PSP was measured retrospectively from stored plasma and CRP was measured routinely, no clear mechanism was identified by which this difference would bias the paired AUROC comparison. The principal concern relates to applicability because the cohort consisted specifically of ED patients with COVID-19.

**Table S1. Comparative risk-of-bias assessment according to QUADAS-C**

| Study | Patient selection | Index tests | Outcome ascertainment | Flow and timing |
| --- | --- | --- | --- | --- |
| García de Guadiana-Romualdo et al., 2019 | Unclear | Low | Low | Low |
| Gukasjan et al., 2013 | Low | High | Unclear | Unclear |
| Melegari et al., 2023 | Unclear | Low | Low | High |
| Michailides et al., 2023 | Low | Low | Unclear | Unclear |
| Que et al., 2012 | Unclear | Unclear | Low | Unclear |
| Que et al., 2015 | Unclear | Unclear | Low | Unclear |
| Van Singer et al., 2021 | Low | Low | Low | Low |

**Legend:** Low, low comparative risk of bias; High, high comparative risk of bias; Unclear, insufficient information to support either a low- or high-risk judgment. QUADAS-C, Quality Assessment of Diagnostic Accuracy Studies–Comparative.

**Table S2. Applicability concerns**

| Study | Population | Index tests | Prognostic outcome |
| --- | --- | --- | --- |
| García de Guadiana-Romualdo et al., 2019 | Low | Low | Low |
| Gukasjan et al., 2013 | High | Low | Low |
| Melegari et al., 2023 | High | Low | Low |
| Michailides et al., 2023 | High | Low | Low |
| Que et al., 2012 | Low | Low | Low |
| Que et al., 2015 | Low | Low | Low |
| Van Singer et al., 2021 | High | Low | Low |

**Legend:** Low, low concern regarding applicability; High, high concern regarding applicability; Unclear, insufficient information to determine applicability.

* Applicability was unclear for PSP measurement and low for CRP/PCT.
† Applicability was unclear for PSP measurement and low for CRP/PCT.

The comparative QUADAS-C assessment showed variation in risk of bias across studies and domains (Figure S1). Patient selection was rated as low risk in three studies and unclear in four. The index-test domain was rated as low risk in four studies, unclear in two, and high risk in one, with the high-risk judgment reflecting differential clinical availability of the compared biomarkers. Outcome ascertainment was generally robust, being rated as low risk in five studies and unclear in two. Flow and timing was rated as low risk in two studies, unclear in four, and high risk in one, mainly reflecting uncertainty regarding biomarker-specific completeness or missing data. Overall, two studies had at least one domain judged at high comparative risk of bias, while no high-risk judgment was assigned to patient selection or outcome ascertainment.

**Figure S1. Comparative risk-of-bias assessment of the included studies using QUADAS-C.** Green (+) indicates low comparative risk of bias, yellow (?) indicates unclear comparative risk of bias, and red (−) indicates high comparative risk of bias. Judgments were based on the review-specific QUADAS-C
